# Cancer-associated fibroblasts promote progression and gemcitabine resistance via the SDF-1/SATB-1 pathway in pancreatic cancer

**DOI:** 10.1038/s41419-018-1104-x

**Published:** 2018-10-18

**Authors:** Lusheng Wei, Huilin Ye, Guolin Li, Yuanting Lu, Quanbo Zhou, Shangyou Zheng, Qing Lin, Yimin Liu, Zhihua Li, Rufu Chen

**Affiliations:** 1 Guangdong Provincial Key Laboratory of Malignant Tumor Epigenetics and Gene Regulation, Sun Yat-sen Memorial Hospital, Sun Yat-sen University, Guangzhou, Guangdong Province China; 2 Department of Pancreatobiliary Surgery, Sun Yat-sen Memorial Hospital, Sun Yat-sen University, Guangzhou, Guangdong Province China; 3 Department of Radiology, Guangzhou women and children’s medical center, Guangzhou Medical University, Guangzhou, Guangdong Province China; 4 Department of Radiotherapy, Sun Yat-sen Memorial Hospital, Sun Yat-sen University, Guangzhou, Guangdong Province China; 5 Department of Medical Oncology, Sun Yat-sen Memorial Hospital, Sun Yat-sen University, Guangzhou, Guangdong Province China

## Abstract

Cancer-associated fibroblasts (CAFs), a dominant component of the pancreatic tumor microenvironment, are mainly considered as promotors of malignant progression, but the underlying molecular mechanism remains unclear. Here, we show that SDF-1 secreted by CAFs stimulates malignant progression and gemcitabine resistance in pancreatic cancer, partially owing to paracrine induction of SATB-1 in pancreatic cancer cells. CAF-secreted SDF-1 upregulated the expression of SATB-1 in pancreatic cancer cells, which contributed to the maintenance of CAF properties, forming a reciprocal feedback loop. SATB-1 was verified to be overexpressed in human pancreatic cancer tissues and cell lines by quantitative real-time PCR, western blot, and immunohistochemical staining, which correlated with tumor progression and clinical prognosis in pancreatic cancer patients. We found that SATB-1 knockdown inhibited proliferation, migration, and invasion in SW1990 and PANC-1 cells in vitro, whereas overexpression of SATB-1 in Capan-2 and BxPC-3 cells had the opposite effect. Immunofluorescence staining showed that conditioned medium from SW1990 cells expressing SATB-1 maintained the local supportive function of CAFs. Furthermore, downregulation of SATB-1 inhibited tumor growth in mouse xenograft models. In addition, we found that overexpression of SATB-1 in pancreatic cancer cells participated in the process of gemcitabine resistance. Finally, we investigated the clinical correlations between SDF-1 and SATB-1 in human pancreatic cancer specimens. In summary, these findings demonstrated that the SDF-1/CXCR4/SATB-1 axis may be a potential new target of clinical interventions for pancreatic cancer patients.

## Introduction

Pancreatic ductal adenocarcinoma (PDAC) is one of the most lethal and aggressive solid malignancies, with a dismal 5-year survival rate of < 7%^1^. In America, PDAC is the fourth leading cause of cancer-related deaths and is expected to become the second leading cause by 2030^2^. The absence of early symptoms and aggressive biological characteristics of tumor are among the reasons for late detection, which makes PDAC act as a silent killer with only 15–20% of cases diagnosed in the early resectable stages^3^. Poor response to available chemotherapy is another main cause of dismal prognosis. In most patients (74%), receiving gemcitabine tumor recurrence is eventually observed, with only 13.4 months of disease-free survival^4^. Better understanding of the complex biological behavior and intricate cellular communication is the prerequisite to developing effective therapeutic strategies.

PDAC is characterized as an abundant desmoplastic tissue that accounts for up to 80% of total tumor mass^5^. This hallmark feature forms the intra-tumoral microenvironment, which consists of the cancer-associated fibroblasts (CAFs), immune cells, capillaries, basement membrane and extracellular matrix (ECM) surrounding the cancer cells^6,7^. CAFs are the most abundant stromal cell type in pancreatic tumor and are characterized by the expression of activation markers, such as α-smooth muscle actin (α-SMA), fibroblast activation protein (FAP), and fibroblast-specific protein 1 (FSP1)^8^. Activated CAFs in PDAC are variously reported to stem from the pancreatic stellate cells, quiescent resident fibroblasts and mesenchymal stem cells. Indeed, CAFs are also derived from epigenetic transitions from endothelial or cancer cells through endothelial–mesenchymal transition or epithelia–mesenchymal transition (EMT)^9,10^. During the progression of CAF activation, the described pathways involve sonic hedgehog, interleukins 6 and 10, transforming growth factor-β1, platelet-derived growth factor (PDGF), basis fibroblast growth factor (bFGF), and other genes^7,8^. CAFs strongly express collagen (type I and III), fibronectin, and hyaluronan, which are the main components of ECM. Increasing evidence indicates that CAFs play an important role in the tumorigenesis, progression, metastasis, and drug resistance^11,12^. However, the biological effects of CAFs on pancreatic cancer progression and chemoresistance remain largely unknown.

Special AT-rich sequence-binding protein 1 (SATB-1) is a nuclear matrix attachment region-binding protein, linking specific DNA elements to its unique cage-like network^13^. SATB-1 can tether genomic loci to the nuclear matrix to form high-order chromatin structure through binding to the AT-rich DNA sequences of base-unpairing regions^14^. SATB-1 also recruits multiple chromatin-modifying enzymes and transcription factors to regulate global gene expression by modifying histones and remodeling nucleosomes^13^. SATB-1 plays a crucial role in the embryonic stem cells and T-cells^15,16^. Han H et al.^17^ were the first to reveal that SATB-1 promoted breast tumor growth and metastasis. Increasing evidence indicated that SATB-1 upregulation was also closely associated with poor prognosis in other malignancies, such as prostate, ovarian, and gastric cancers, as well as in hepatocellular and renal cell carcinomas^18–25^. Elevated expression of SATB-1 was also associated with poor prognosis in pancreatic cancer^26,27^. However, the specific roles of SATB-1 in CAFs promoted pancreatic cancer progression are poorly elucidated.

In this study, we show that SDF-1, a characteristic C-C chemokine released by tumor-associated fibroblasts, can prominently upregulate the expression of SATB-1 and subsequently contribute to malignant progression and gemcitabine resistance of pancreatic cancer cells. In addition, we have also found that overexpression of SATB-1 in pancreatic cancer cells in turn plays a vital role in maintaining the local supportive function of CAFs, indicating the formation of a SATB-1-centered positive feedback loop in pancreatic cancer. Finally, we examined the clinical correlation of SATB-1 and SDF-1 in human pancreatic cancer specimens. Taken together, our present work provides solid evidence for reciprocal interactions between CAFs and pancreatic cancer cells, shedding new light on the utilization of the SDF-1/CXCR4/SATB-1 axis as a potential therapeutic target for the treatment of pancreatic cancer.

## Results

### Characterization of primary NAFs and CAFs

**Fig. 1:**
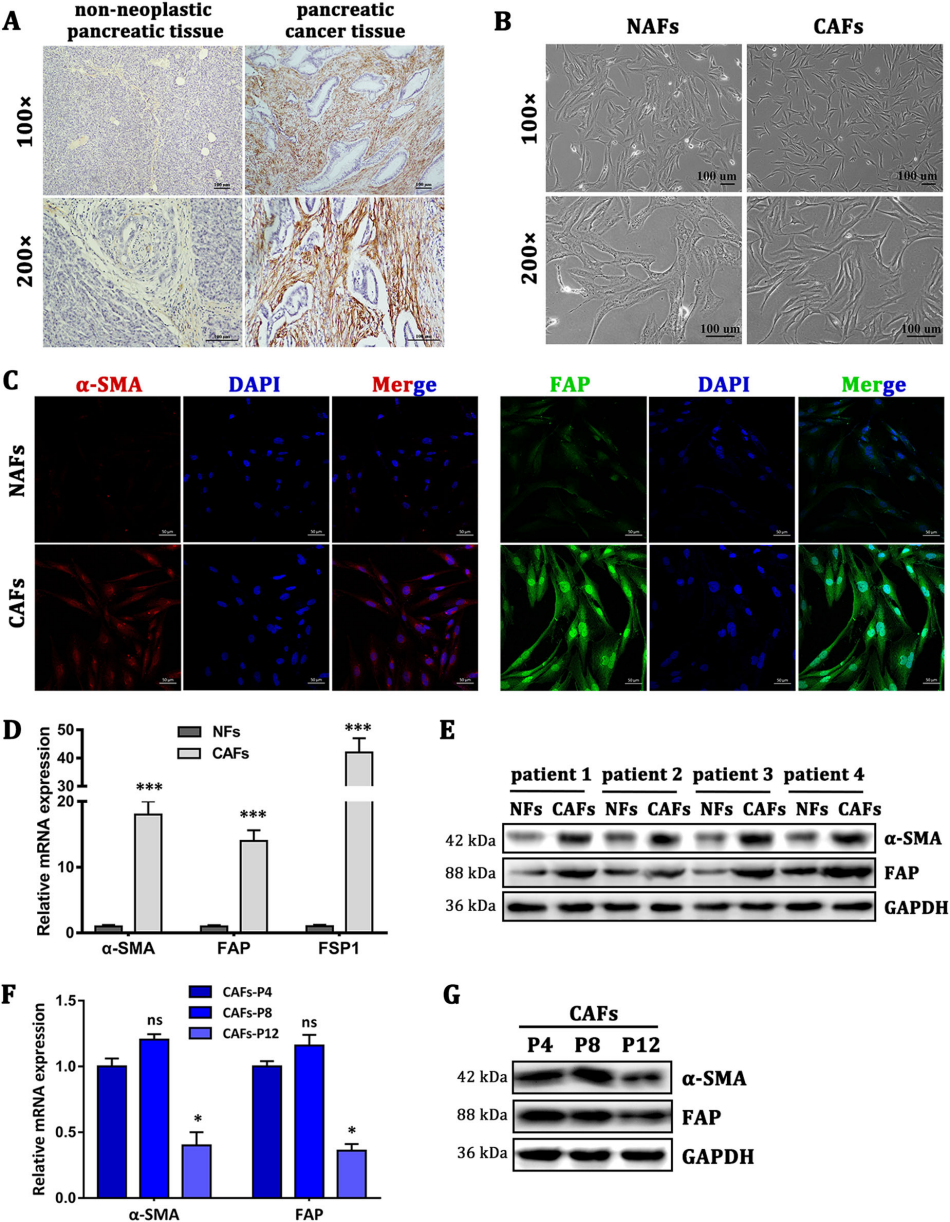
Characterization of primary NAFs and CAFs **a** The expression of α-SMA in tumor stroma was assayed by immunohistochemical staining, indicating that CAFs were abundant in pancreatic tumor stroma. **b** The morphological images of NAFs and CAFs. **c** Immunofluorescence staining showed the subcellular localization and the expression of α-SMA and FAP in NAFs and CAFs. Scale bar = 50 μm, magnification,  ×400. **d** The mRNA expression levels of α-SMA, FAP, and FSP1 in NAFs and CAFs (passage 4) isolated from three patients were detected by qRT-PCR analysis. *n* = 3 (replicating from three patients), ^***^p < 0.001. **e** Western blot analysis shows the expression of α-SMA and FAP in NAFs and CAFs derived from four pairs of non-neoplastic pancreatic tissues and tumor tissues. **f**, **g** qRT-PCR and western blot analysis show the different expression levels of α-SMA and FAP in CAFs at different passages. Compared with the fourth-passage CAFs (CAFs-P4), the expression of α-SMA and FAP in CAFs-P8 had no significant difference, but the expression in CAFs-P12 significantly decreased. *n* = 3 (replicating from three patients), ns: not significantly different, ^*^p < 0.05

The human pancreatic normal tissue-associated fibroblasts (NAFs) and CAFs were isolated from fresh pancreatic cancer tissue and adjacent non-neoplastic pancreatic tissue, further confirmed with postoperative cancer tissue by immunohistochemistry staining of α-SMA (Fig. 1a). The primary fibroblasts initially showed bi- and/or multipolar morphology (Fig. 1b). As showed in Fig. 1c, the results of cell immunofluorescence staining indicated that CAFs highly expressed α-SMA and FAP, which were not or weakly expressed in NAFs. NAFs and CAFs both expressed vimentin but not cytokeratin 19 (Supplementary Figure 1). To test the purity of NAFs and CAFs, fibroblast biomarkers were examined by quantitative real-time PCR (qRT-PCR), western blot, and immunofluorescence staining. Compared with NAFs, CAFs had high mRNA expression levels of FAP, FSP1, and α-SMA (Fig. 1d). Comparing NAFs and CAFs derived from four PDAC patients we detected the protein expression levels of α-SMA and FAP to further verify their utility as biomarkers (Fig. 1e). Moreover, we found out that CAFs would be in a worse growth status if CAFs were digested and passed for too many generations. Loss of CAFs markers was also found in CAFs derived from gastric carcinoma^28^. Lack of tumor-secreted factors induction may cause the downregulation of CAFs markers. Then, we performed qRT-PCR and western blot analyses to compare the markers’ expression of CAFs at different passages. The results showed that the twelfth-passage CAFs produced lower protein expression of α-SMA and FAP compared with the fourth-passage CAFs (Fig. 1f, g). Altogether, these data indicated that we successfully isolated high-purity NAFs and CAFs from PDAC specimens.

### SATB-1 was overexpressed in pancreatic cancer cell lines and tissue samples and upregulated by CAFs

**Fig. 2:**
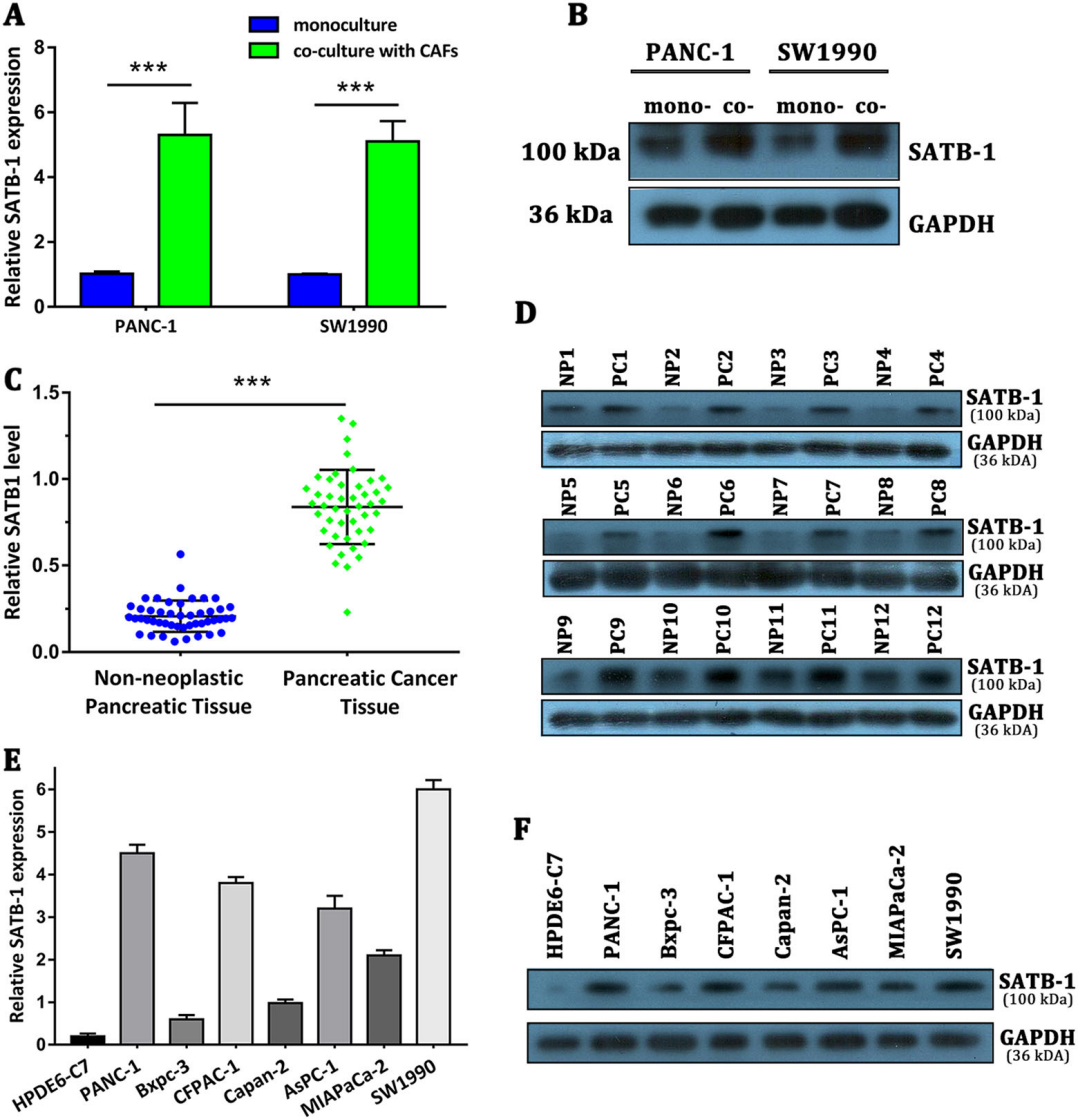
SATB-1 is overexpressed in pancreatic cancer cell lines and tissue samples and is upregulated by CAFs **a**, **b** The qRT-PCR and western blot analysis show the mRNA and protein levels of SATB-1 in SW1990 and PANC-1 cells cultured with (co-culture) or without CAFs (monoculture). *n* = 3, ^***^p < 0.001. **c** The qRT-PCR analysis shows the mRNA expression of SATB-1 in 32 pancreatic tumor tissues and matched non-neoplastic pancreatic tissues. Dots represent each patient, and error bars indicate standard deviation (SD). *n* = 3, ^***^p < 0.001. **d** Western blot analysis shows the protein levels of SATB-1 in 12 pancreatic cancer tissues (PC) and matched non-neoplastic pancreatic tissues (NP). **e**, **f** The qRT-PCR and western blot analyses show the mRNA and protein levels of SATB-1 in various pancreatic cancer cell lines. *n* = 3

As CAFs demonstrated potent protumor properties in pancreatic cancer, we aimed to characterize the downstream molecular events responsible for CAF-mediated malignant progression of pancreatic cancer using the Human 12 × 135 K Gene Expression Array manufactured by Roche NimbleGen. Using the transwell co-culture system, we co-cultured PANC-1 pancreatic cancer cells and NAFs or CAFs for 4 days, and subsequently investigated the differential expression profiles of mRNA in PANC-1 cells between the PANC-1 co-cultured with NAFs group (PCN group) and the PANC-1 co-cultured with CAFs group (PCC group). A total of 45,033 mRNA targets were detected by microarray probes in two groups of samples. Overall, 170 mRNAs were evaluated to be differentially modulated by fold change > 2.0, with the *p* value < 0.05 and false discovery rate < 0.05 (PCC group vs. PCN group). Among them, 131 mRNAs were upregulated, whereas 39 mRNAs were downregulated. In the present study, the top 10 upregulated mRNAs are listed by fold change, among which the cell adhesion molecule SATB-1 was the fourth upregulated gene with ~ 4.8-fold change (Table 1). Aberrant expression of SATB-1 has been reported in cells of various cancer types, including prostate cancer, ovarian cancer, gastric cancer, hepatocellular carcinoma, renal cell carcinoma, rectal cancer, bladder cancer, laryngeal squamous cell carcinoma, and cutaneous malignant melanoma^29,30^. However, the specific role of SATB-1 in pancreatic cancer remains elusive. Hence, we chose SATB-1 as a gene of interest and set out to determine whether SATB-1 facilitates malignant progression of pancreatic cancer and participates in the crosstalk between tumor cells and CAFs. qRT-PCR and western blotting showed that SATB-1 was upregulated in PANC-1 and SW1990 pancreatic cancer cells only when co-cultured with CAFs, validating our microarray results (Fig. 2a, b). To investigate SATB-1 mRNA expression levels in PDAC, we performed qRT-PCR analysis on total RNA extracted from 46 pancreatic cancerous tissues and their matched non-neoplastic counterparts. Our current results showed that SATB-1 mRNA was significantly overexpressed in pancreatic cancerous samples in comparison with those of corresponding normal tissues (Fig. 2c). Subsequently, we randomly selected 12 paired PDAC samples to evaluate the SATB-1 protein expression level using western blot analysis. In agreement with the PCR observations, the SATB-1 protein level was significantly upregulated in pancreatic cancerous tissues (Fig. 2d). Moreover, seven PDAC cell lines (PANC-1, SW1990, Capan-2, CFPAC-1, BxPC-3, AsPC-1, and MIAPaCa-2) also showed significantly higher SATB-1 mRNA and protein levels than the pancreatic ductal epithelium cell line HPDE6-C7, with the two highest expression levels observed in PANC-1 and SW1990 cells (Figure E-F). Although Capan-2 and Bxpc-3 expressed lower SATB-1, they would also upregulate the SATB-1 expression in CAFs co-culture system (Supplementary Figure 2). In summary, these results manifested that CAFs can significantly upregulate the expression of SATB-1 in pancreatic cancer cells and that SATB-1 is markedly increased in both pancreatic cancerous tissues and pancreatic cancer cell lines.

**Table 1:** Top 10 upregulated mRNAs in PANC-1 cells co-cultured with CAFs

Fold change	*P* value	Regulation	Gene Symbol	Description
5.7183622	0.000441	Up	OR9G1	Olfactory receptor family 9 subfamily G member 1
5.3407117	0.035397	Up	RASAL3	RAS protein activator like 3
4.9443596	0.029836	Up	ARHGAP30	Rho GTPase activating protein 30
4.8004442	0.014924	Up	SATB-1	SATB homeobox 1
4.3003547	9.53E-06	Up	TJP3	Tight junction protein 3
4.1499271	1.45E-06	Up	SH2D3C	SH2 domain containing 3C
3.8759635	0.032572	Up	FNDC3A	Fibronectin type III domain containing 3A
3.7832849	0.020696	Up	DEF6	DEF6, guanine nucleotide exchange factor
3.6076283	0.010234	Up	C19orf45	Chromosome 19 open reading frame 45
3.5313205	0.005514	Up	IP6K3	Inositol hexakisphosphate kinase 3

### SDF-1 secretion is related to CAF-induced SATB-1 upregulation in pancreatic cancer cells

**Fig. 3:**
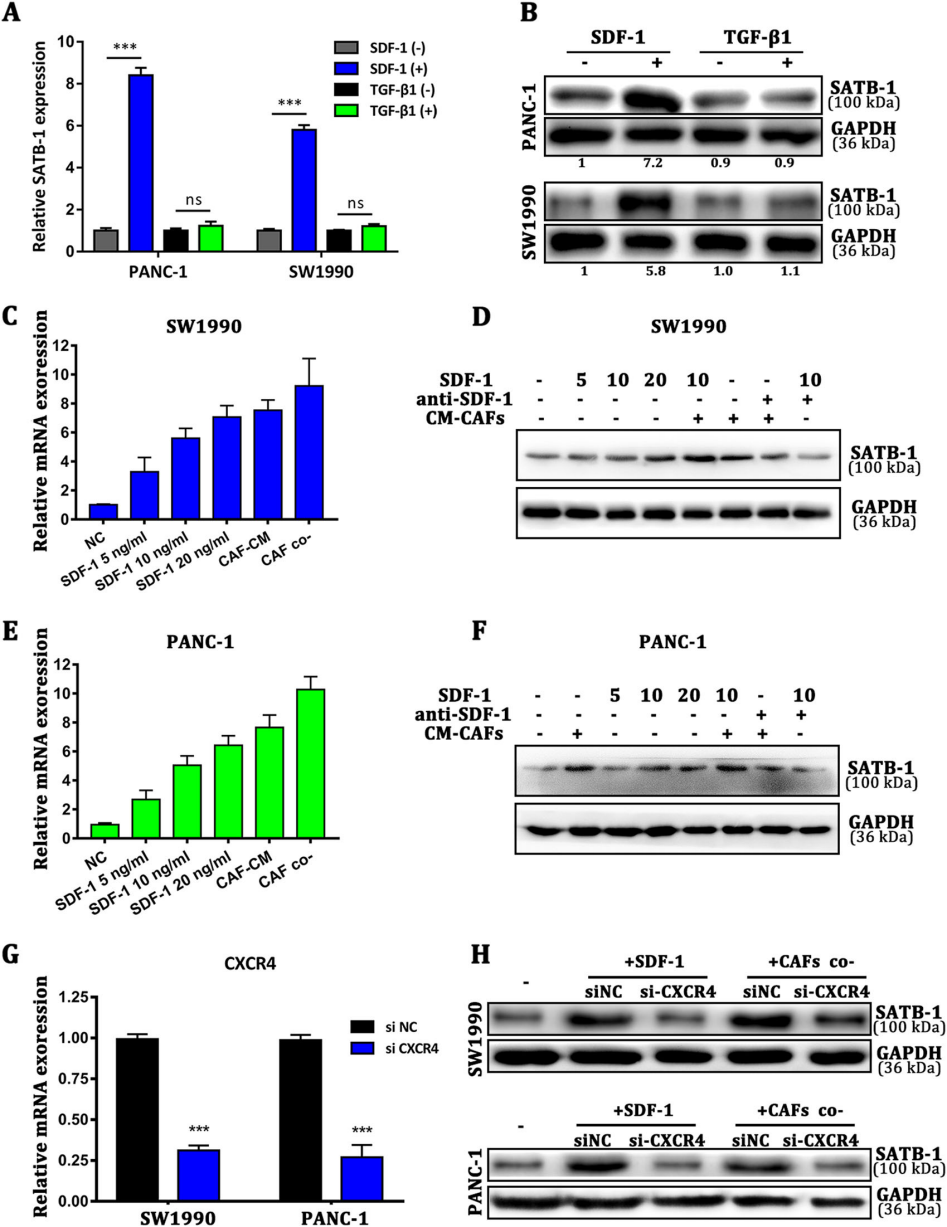
SDF-1 secretion is related to CAF-induced SATB-1 upregulation in pancreatic cancer cells **a**, **b** The qRT-PCR and western blot analysis show the mRNA expression and protein levels of SATB-1 in SW1990 and PANC-1 cells after the induction of SDF-1 and TGF-β1. *n* = 3, ns: not significantly different, ^***^p < 0.001. **c**, **d** The qRT-PCR and western blot analyses show the mRNA and protein levels of SATB-1 in SW1990 cells with the addition of various concentrations of SDF-1 and CM-CAFs. **e**, **f** The qRT-PCR and western blot analyses show the mRNA and protein levels of SATB-1 in PANC-1 cells with the addition of various concentration of SDF-1, CM-CAFs, or anti-SDF-1 (4 μg/ml). **g** The qRT-PCR analysis indicates that the expression of CXCR4 in SW1990 and PANC-1 cells was effectively downregulated. *n* = 3, ^***^p < 0.001. **h** The western blot analysis shows the protein levels of SATB-1 in SW1990 and PANC-1 cells with CXCR4 silenced. SW1990 and PANC-1 cells were incubated with SDF-1 for 4 days or co-cultured with CAFs for 4 days

In many cocultures with different types of tumor cells, CAFs enhanced tumorigenesis, metastasis, or drug resistance of cancer cells via secretion of many soluble factors^9^. Then, we analyzed the mRNA expression of PGDF-A, VEGF, IGF-1, hepatocyte growth factor (HGF), bFGF, SDF-1, IL-6, CCL18, TNF-α, and TGF-β1 of NAFs and CAFs (Supplementary Figure 3A). We found that TGF-β1 and SDF-1 were two of the highest cytokines secreted by CAFs, similar to previous study^31^. Then, we performed enzyme-linked immunosorbent assay (ELISA) analysis to further confirm the secretion of TGF-β1 and SDF-1 in CAFs-CM compared with matched NAFs-CM (Supplementary Figure 3B). CAFs-secreted cytokines may promote the expression of SATB-1 in pancreatic cancer cells. Therefore, we analyzed the mRNA expression of SATB-1 in PANC-1 and SW1990 cells after the induction of human recombinant TGF-β1 and SDF-1. We found that recombinant SDF-1 upregulated SATB-1 expression 3.28-fold, whereas recombinant TGF-β1 produced no change (Fig. 3a, b). Previous studies have reported that CXCR4 is the main cognate receptor of SDF-1, and the SDF-1/CXCR4 biological axis has an important role in many types of solid tumors, including pancreatic, breast, gastric, and lung cancers. Therefore, we examined whether the SDF-1/CXCR4 axis regulates the SATB-1 expression level in pancreatic cancer cells. We analyzed the SATB-1 expression level in SW1990 and PANC-1 cells after an addition of human recombinant SDF-1 or conditioned medium derived from CAFs (CM-CAFs), or after indirect coculturing with CAFs. The mRNA expression level of SATB-1 was upregulated by SDF-1, CM-CAFs, and CAFs (Fig. 3c). The western blot data showed a similar result (Fig. 3d). Moreover, when SDF-1 was neutralized, the level of SATB-1 protein in SW1990 and PANC-1 cells similar to that in SW1990 and PANC-1 cells cultured alone. On the other hand, when silencing CXCR4 with siRNA before treating cells with SDF-1, the effect of SDF-1 on upregulation of the SATB-1 expression level was abrogated, as evidenced by the return of SATB-1 mRNA and protein level to the baseline level (Figure 3g–h). In conclusion, these data demonstrated that CAFs upregulated the expression level of SATB-1 in pancreatic cancer cells through the SDF-1/CXCR4 axis.

### SATB-1 mediates the proliferation and G1-S checkpoint of pancreatic cancer cells in vitro

**Fig. 4:**
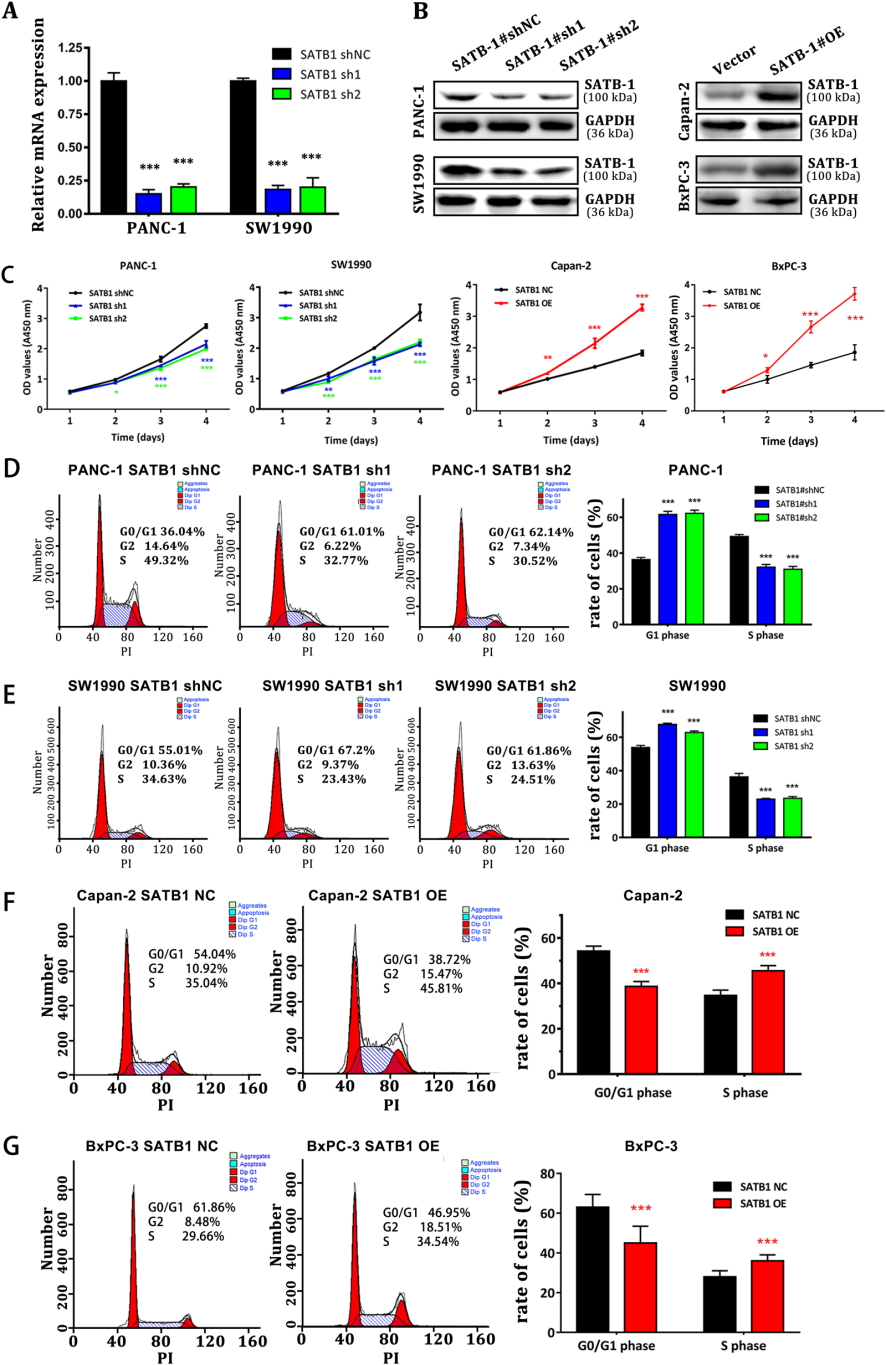
SATB-1 mediates the proliferation and G1-S checkpoint of pancreatic cancer cells in vitro **a**, **b** The qRT-PCR and western blot analyses show that the expression levels of SATB-1 in PANC-1 and SW1990 cells with specific sh-RNA were downregulated. SATB-1 was overexpressed at the protein level in Capan-2 and BXPC-3 cells transfected with pcDNA3.1-SATB-1. *n* = 3, ^***^p < 0.001. **c** CCK-8 analysis shows the growth curve of PANC-1 and SW1990 cells with SATB-1 stably silenced and the growth curve of Capan-2 and BXPC-3 cells overexpressing SATB-1. *n* = 3, ^*^p < 0.05, ^**^p < 0.01, ^***^p < 0.001. **d**, **e** FACS analysis shows the impact of SATB-1 on cell cycle distribution of PANC-1 and SW1990. *n* = 3, ^***^p < 0.001. **f**–**g** FACS analysis shows the impact of SATB-1 on cell cycle distribution of Capan-2 and BXPC-3 cells. *n* = 3, ^***^p < 0.001

To further investigate the impact of SATB-1 on pancreatic cancer progression, in vitro functional characterizations were performed. First, qRT-PCR assay and western blot analysis indicated that SATB-1 expression was significantly reduced by specific small interfering RNAs (siRNAs) in SW1990 and PANC-1 cells and upregulated after transfection of pcDNA3.1-SATB-1 cDNA vector into Capan-2 and BXPC-3 cells (Fig. 4a, b). Compared with control cells, SATB-1 downregulation significantly inhibited tumor cell proliferation in SW1990 and PANC-1 cells, as shown by the CCK-8 assay. In contrast, SATB-1 overexpression had an opposite effect (Fig. 4c). In addition, we performed the flow cytometry analysis for the distribution of cell cycle. The results demonstrated that downregulation of SATB-1 led to a substantial accumulation of PDAC cells in G0/G1 phase, accompanied by a substantial decrease in S phase (Fig. 4d, e). SATB-1 overexpression, however, had an opposite influence on the cell cycle distribution in Capan-2 and BxPC-3 cells (Fig. 4f, g). Interestingly, SATB-1 downregulation in PDAC cells made no difference in the fraction of apoptotic cells (Supplementary Figure 4). Altogether, SATB-1-induced acceleration of PDAC cell proliferation appeared to be facilitated by variation of the G1-S checkpoint, rather than by cell apoptosis.

### SATB-1 promotes PDAC cell migration and invasion in vitro

**Fig. 5:**
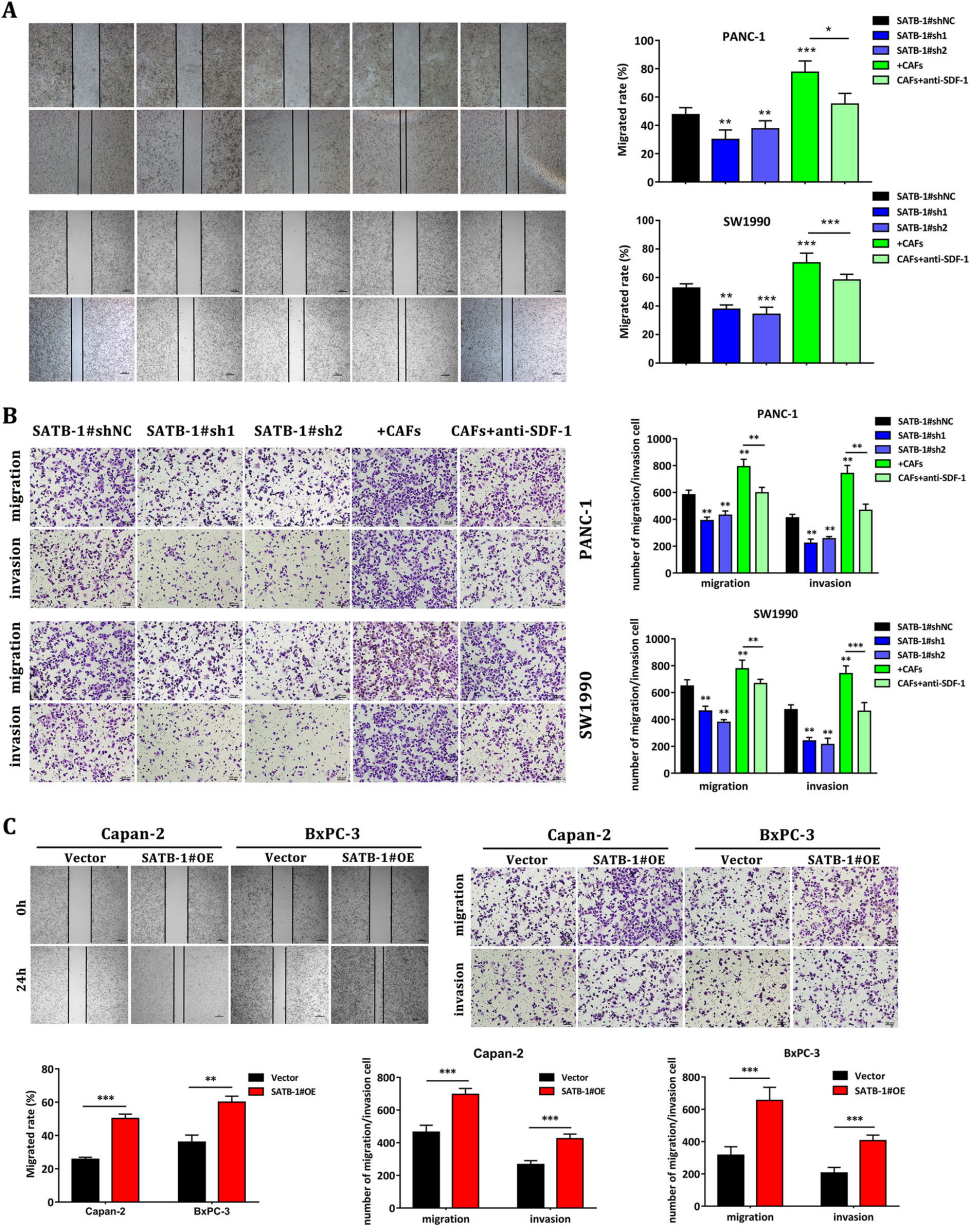
SATB-1 enhanced the migration and invasion abilities of pancreatic cancer cells **a** Wound-healing assay shows the abilities of SW1990 and PANC-1 cells with SATB-1 silenced or co-cultured. **b** The transwell assay shows the fractions of migrated and invaded SW1990 and PANC-1 cells with SATB-1 silenced or co-cultured. Knockdown of SATB-1 inhibited the migration and invasion abilities of PANC-1 and SW1990 cells. Coculturing with CAFs enhanced the migration and invasion abilities of PANC-1 and SW1990 cells, but neutralization with anti-SDF-1 antibody reduced these abilities. Compared with control pancreatic cancer cells, *n* = 3, ^*^p < 0.05, ^**^p < 0.01, ^***^p < 0.001. **c** Wound-healing and transwell assays show the upregulated migration and invasion abilities of Capan-2 and BXPC-3 cells transfected with pcDNA3.1-SATB-1. Compared with control pancreatic cancer cells, *n* = 3, ^**^p < 0.01, ^***^p < 0.001

Enhanced cell migration and invasion abilities underlie the mechanisms of cancer metastasis, resulting in poor prognosis. Our wound-healing scratch assay clarified that SATB-1 knockdown in PANC-1 and SW1990 cells markedly decreased cell motility compared with the si-NC control groups, whereas the coculturing with CAFs promoted the capability of healing. Then, the wound-healing postponed when anti-SDF-1 antibodies neutralized the SDF-1 derived from CAFs in the co-culture system (Fig. 5a). Further, the transwell assay demonstrated that downregulation of SATB-1 prominently weakened the migration and invasion of PANC-1 and SW1990 cells, whereas CAFs strengthened these capabilities of PANC-1 and SW1990 cells (Fig. 5b). In contrast, overexpression of SATB-1 significantly enhanced the migration and invasion of Capan-2 and BxPC-3 cells (Fig. 5c), as indicated by wound-healing and transwell analyses. These observations verified that SATB-1 enhanced PDAC cell migration and invasion via CAF-secreted SDF-1 in vitro.

### Overexpression of SATB-1 in pancreatic cancer cells reciprocally sustains the hallmark of CAFs in vitro

**Fig. 6:**
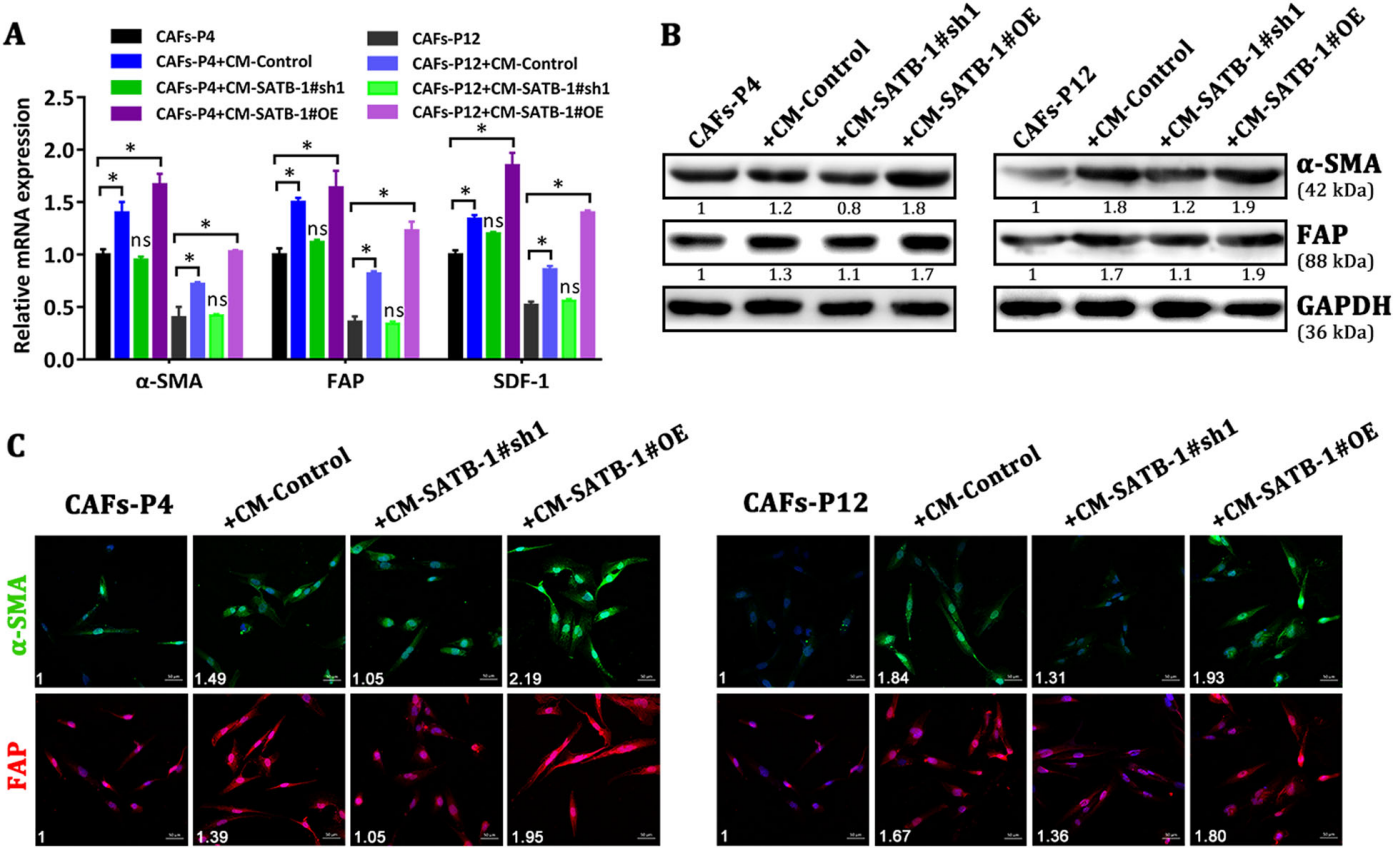
Overexpression of SATB-1 in pancreatic cancer cells sustains the hallmark of CAFs in vitro **a** qRT-PCR assay shows the expression of α-SMA, FAP, and SDF-1 in CAFs-P4 and CAFs-P12 induced with conditioned media from control SW1990 cells, SATB-1-silenced SW1990 cells or SATB-1-overexpressed BXPC-3 cells. *n* = 3, ns: not significantly different, ^*^p < 0.05. **b**, **c** Western blot and IFC staining show the expression of α-SMA and FAP in CAFs-P4 and CAFs-P12 induced with conditioned medium from control SW1990 cells, SATB-1-silenced SW1990 cells or SATB-1-overexpressed BxPC-3 cells. After induction with conditioned media from SW1990-Control or BxPC-3-SATB-1#OE cells, CAFs expressed higher levels of α-SMA and FAP. Conditioned medium from SW1990-SATB-1#sh1 cells upregulated the expression of α-SMA and FAP in CAFs

As CAFs exert significant impact on the metastatic hallmark of pancreatic cancer, we wondered whether overexpression of SATB-1 in pancreatic cancer cells could influence the fibroblast phenotype. We cultured CAFs with CM from either control pancreatic cancer cells or SATB-1-silenced pancreatic cancer cells and analyzed the α-SMA and FAP expression levels in CAFs. First, qRT-PCR assay indicated that the mRNA expression of α-SMA, FAP, and SDF-1 was significantly increased in CAFs-P4 and CAFs-P12 induced with CM from SW1990-control cells and SATB-1-overexpressed BxPC-3 cells compared with control CAFs (Fig. 6a). However, conditioned medium from SATB-1-silenced SW1990 cells did not increase the expression of hallmark mRNAs in CAFs-P4 and CAFs-P12. Moreover, western blot and immunocytochemistry staining further confirmed the above results (Fig. 6b, c). Then, NAFs were also treated with conditioned medium from pancreatic cancer cells with different SATB-1 expression. The result indicated that SATB-1 expression was also involved in the activation of CAFs from NAFs (Supplementary Figure 5). These data suggest that overexpression of SATB-1 in pancreatic cancer cells reciprocally sustains CAF-like features and contributes to the malignant characteristics of the fibroinflammatory tumor microenvironment.

### SATB-1 mediates gemcitabine resistance in PDAC cells in vitro

**Fig. 7:**
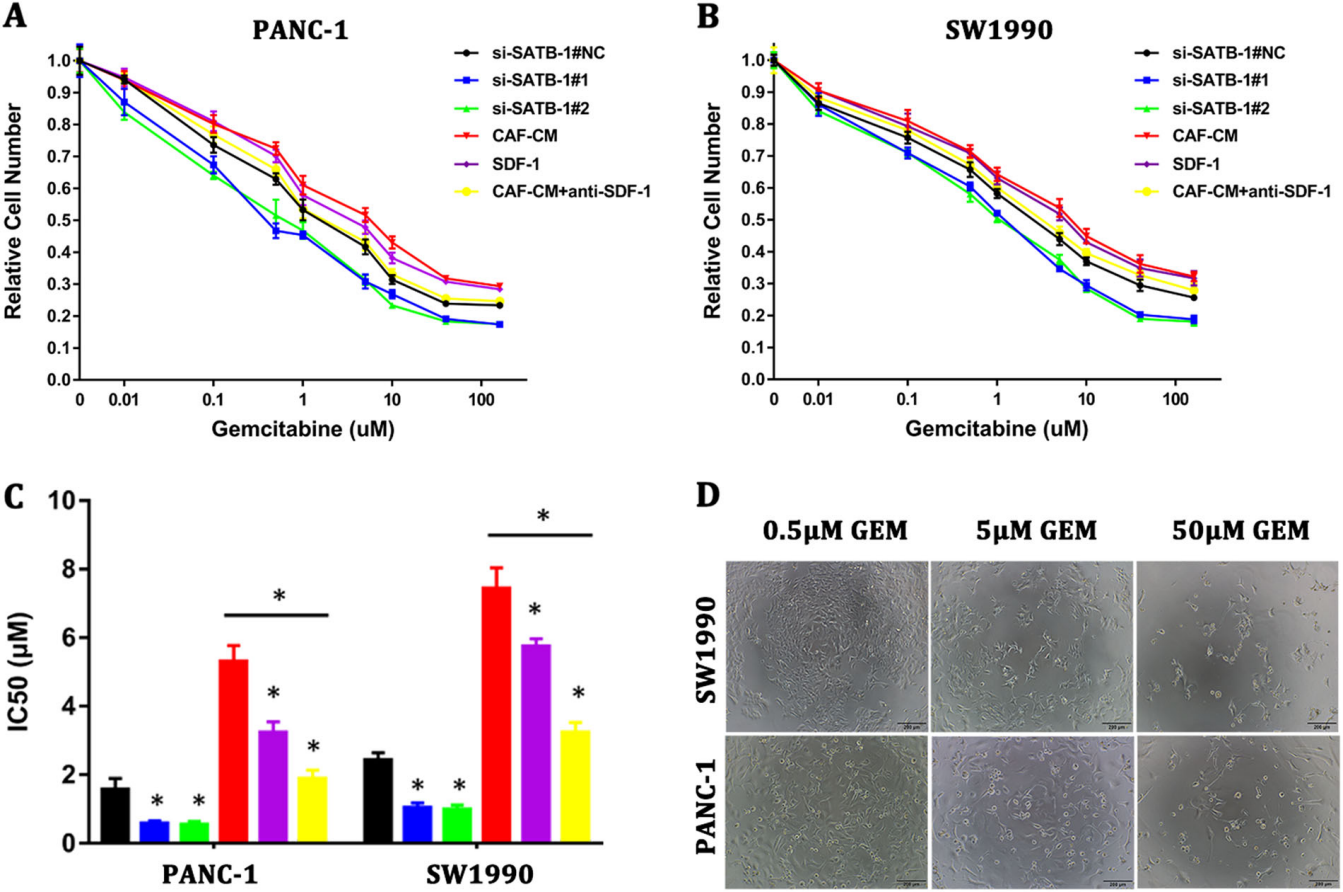
SATB-1 mediated gemcitabine resistance in pancreatic cancer cells in vitro **a**, **b** CCK-8 assay showed the survival rate of PANC-1 and SW1990 cells induced with various concentrations of gemcitabine in different conditions. *n* = 3. **c** The chart shows the IC50 values for PANC-1 and SW1990 cells induced with various concentrations of gemcitabine in different conditions. *n* = 3, *p < 0.05. **d** The images show the morphological changes of PANC-1 and SW1990 cells induced with various concentrations of gemcitabine

Resistance to anticancer drugs critically limits the effectiveness of chemotherapy. Gemcitabine is the standard chemotherapy drug for pancreatic cancer^32^. To investigate whether overexpression of SATB-1 in PDAC cells correlates with an active role in gemcitabine resistance, we measured the half-maximal inhibitory concentration (IC50) of gemcitabine in SW1990 and PANC-1 cells under various conditions (Fig. 7a, b). Downregulation of SATB-1 decreased the IC50 values in pancreatic cancer cells, thus indicating that SATB-1 played an active role in promoting gemcitabine resistance in pancreatic cancer cells. Moreover, an addition of both exogenous recombinant SDF-1 and conditioned medium derived from CAFs (CAFs-CM) increased the IC50 values, whereas the promoting effect of CAFs-CM was not observed in the presence of a neutralizing antibody against SDF-1. The IC50 values were calculated as shown in Fig. 7c. We further observed the morphological changes of SW1990 and PANC-1 incubated with gemcitabine at various concentrations, and the surviving cells came shrinking with elongated pseudopodiums (Fig. 7d). Together with the data presented above, these results suggested that CAFs induced the gemcitabine resistance in pancreatic cancer cells by upregulation of SATB-1 via CAF-secreted SDF-1.

### SATB-1 is important for pancreatic tumor growth in vivo

**Fig. 8:**
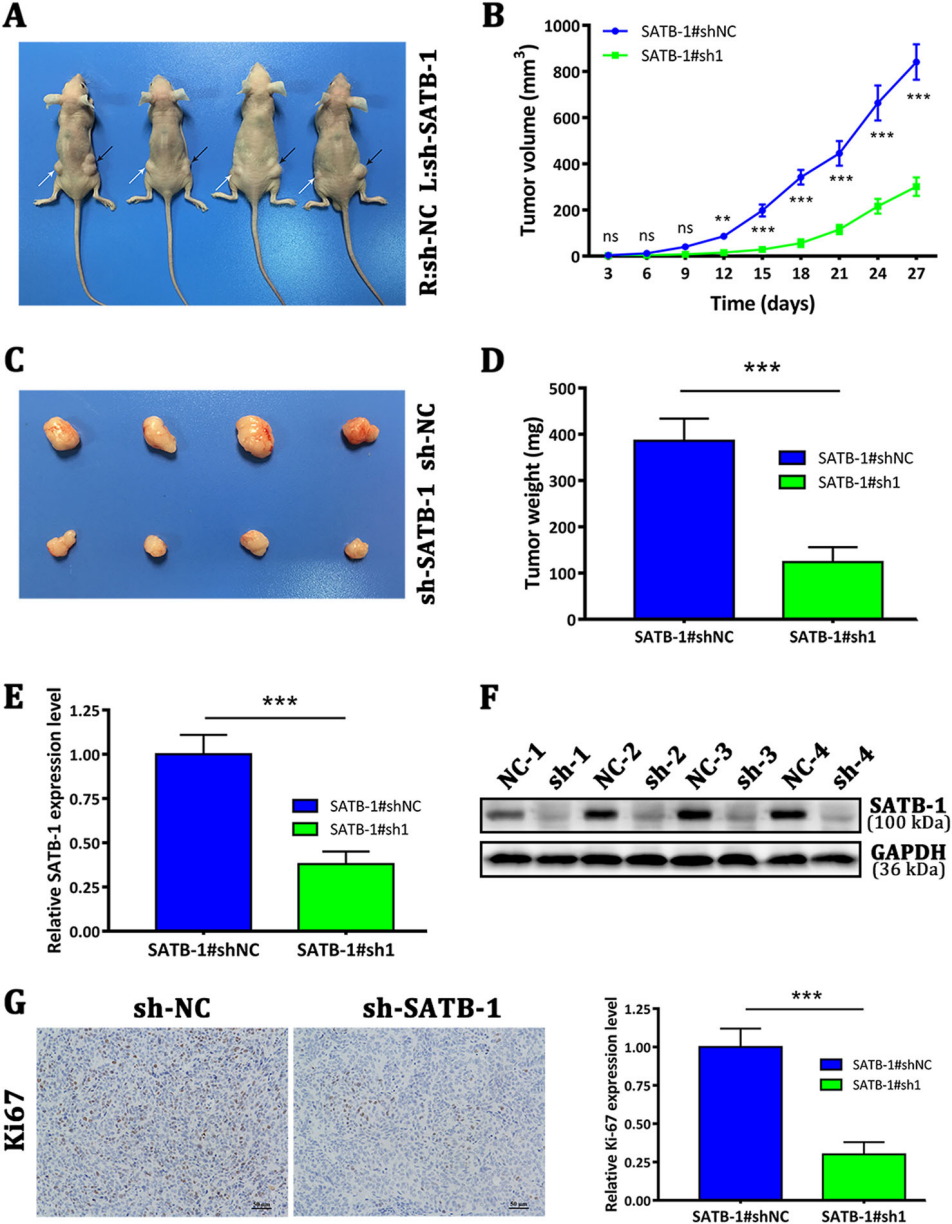
Knockdown of SATB-1 inhibits tumor growth in mouse xenograft models **a** SW1990 cells with a stable SATB-1 knockdown or mock cells were subcutaneously injected into the bilateral hind legs of nude mice. At 27 days after injection, SW1990 cells transfected with sh-SATB-1 (white arrow) and mock cells (black arrow) developed primary tumors. **b** Tumor growth curve. The points and bars represent means ± SD. *n* = 4, ns: not significantly different. ^**^p < 0.01, ^***^p < 0.001. **c** The images of harvested tumors. **d** Tumor weights are shown as means ± SD. *n* = 4, ^***^p < 0.001. **e**, **f** The qRT-PCR and western blot assay analyzed the mRNA and protein expression levels of SATB-1 in tumor tissues from sh-SATB-1 SW1990 cells compared with sh-NC SW1990 cells. **g** IHC staining showed the reduced expression of Ki-67, a proliferation marker, in tumor samples from sh-SATB-1 SW1990 cells. *n* = 4, ^***^p < 0.001

To investigate whether SATB-1 played a role in tumor formation in vivo, we performed a subcutaneous injection of SW1990 cells with stable SATB-1 knockdown (SATB-1#sh1) or mock-transfected cells (sh-NC) into the bilateral hind legs of athymic nude mice. A month later, all mice developed xenograft tumors at the injection site (Fig. 8a, b). By qRT-PCR analysis and western blot assay, we confirmed the SATB-1 knockdown in the xenotransplanted tumors in the sh-SATB-1 group (Fig. 8e, f). Immunostaining showed that tumors in the sh-SATB-1 group inhibited positive expression of Ki-67 compared with the sh-NC group (Fig. 8g). Consistent with the in vitro results, xenograft tumors grown in SATB-1-silenced SW1990 cells had a smaller mean size and weight than xenograft tumors grown in mock cells (Fig. 8c, d). The growth of implanted tumor with SW1990-SATB-1#sh2 was inhibited (Supplementary Figure 6). Taken together, these findings indicated that SATB-1 played a vital role in PDAC proliferation capacity in mouse xenograft models.

### SDF-1 and SATB-1 overexpression correlates with clinicopathological characteristics and poor prognosis of PDAC patients

**Fig. 9:**
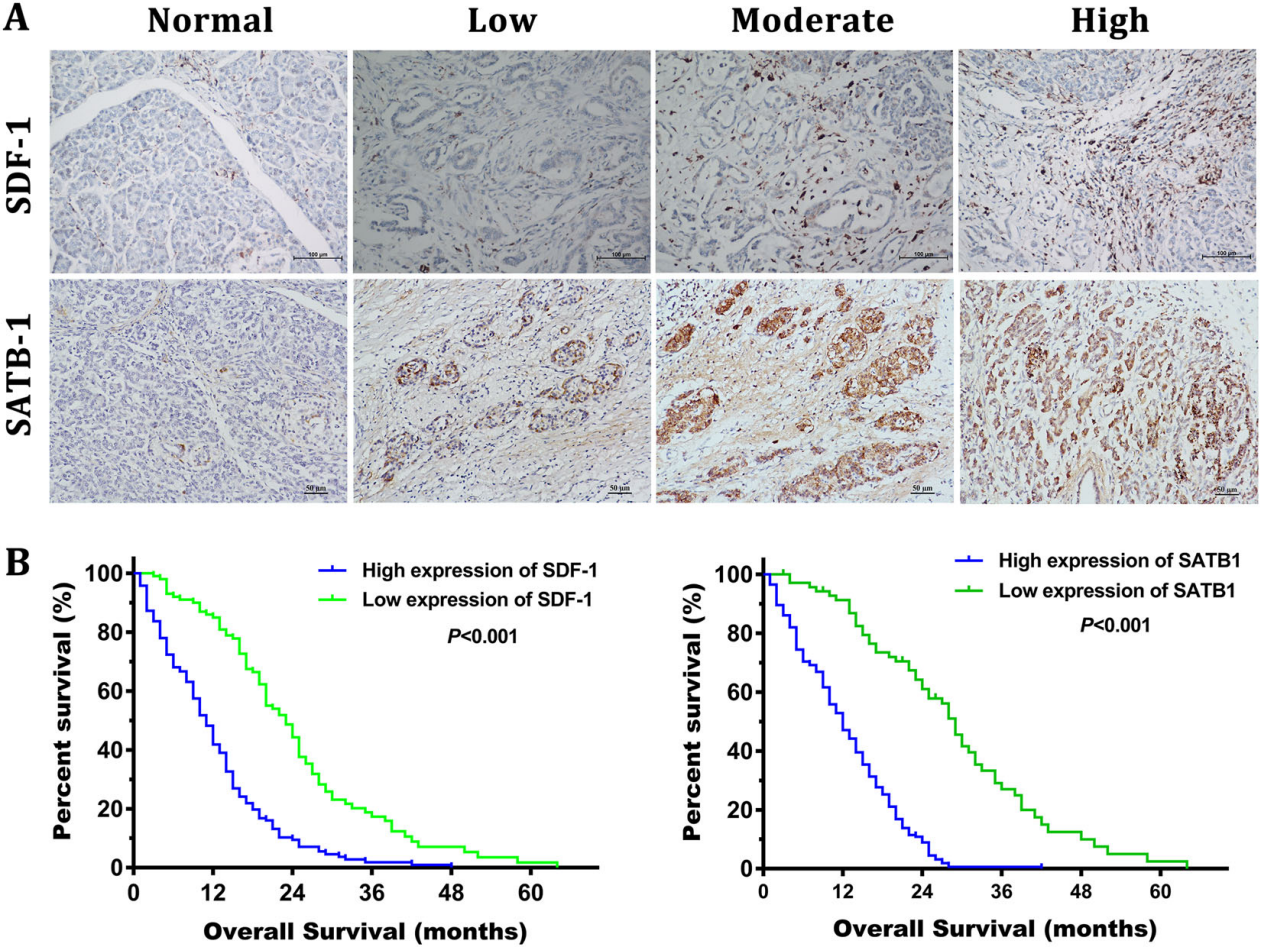
Overexpression of SDF-1 and SATB-1 is correlated with clinicopathological characteristics and poor prognosis of PDAC patients **a** Representative images of SDF-1 and SATB-1 staining in PDAC tissue (intensity: low, moderate, and high). The expression of SDF-1 and SATB-1 were evaluated semi-quantitatively based on staining intensity and distribution according to immunoreactive score. **b** The Kaplan–Meier analysis of overall survival of PDAC patients stratified by the SDF-1 or SATB-1 immunoreactive scores. The log-rank test was performed to compare differences between groups

To investigate the clinical relevance of the SDF-1/SATB-1 axis in pancreatic cancer, we analyzed the expression levels of SDF-1 and SATB-1 protein in 243 paraffin-embedded human PDAC samples by immunohistochemistry. As described in the Methods, the expression levels of SDF-1 and SATB-1 were separately evaluated according to the intensity and percentage and finally stratified into two groups (low and high expression groups; score range of 0–12). Figure 9a shows representative images of these two groups. The relationship between the intensity of SDF-1 or SATB-1 staining and clinicopathological features was also analyzed. Statistical analysis confirmed that SDF-1 overexpression only correlated with the lymph node metastasis, whereas SATB-1 overexpression correlated with differentiation, T stage, TNM stage, and lymph node metastasis (Table 2). Furthermore, the Kaplan–Meier survival plots displayed that higher SDF-1 or SATB-1 staining intensity correlated with poorer prognosis in PDAC patients (log-rank test, *p* *<* 0.001, Fig. 9b). Moreover, the univariate analysis showed that tumor differentiation, T stage, TNM stage, lymph node metastasis, SATB-1 expression, and SDF-1 expression were significantly associated with an increased risk of cancer-related death. The multivariate analysis indicated that T stage, TNM stage, lymph node metastasis, SATB-1 expression and SDF-1 expression were independent prognostic factors (Table 3). To investigate the relationship between SDF-1 expression and SATB-1 expression, Chi-Square test was used to analyze and the result showed that those patients with higher SDF-1 expression tended to express higher SATB-1 (*p* < 0.05) (Table 4).

**Table 2:** Correlation between SATB-1 and SDF-1 expression and clinicopathological characterics of PDAC patients

Characteristics	N of cases	SATB-1 level			*SDF-1 level*		
		H	L	*P* value	H	L	*P v*alue^a^
Total cases	243	172	71		141	102	
Sex							
Male	142	101	41	0.889	80	62	0.528
Female	101	71	30		61	40	
Age							
< 60	104	76	28	0.496	61	43	0.864
≥ 60	139	96	43		80	59	
Differentiation							
Poor	68	58	10	0.008^**^	46	22	0.158
Moderate	118	78	40		65	53	
Well	57	36	21		30	27	
T stage							
T1	4	2	2	0.003^**^	2	2	0.303
T2	28	15	13		14	14	
T3	176	122	54		100	76	
T4	35	32	3		25	10	
TNM stage (AJCC)^b^							
I	17	10	7	0.025^*^	10	9	0.057
II	130	88	57		72	58	
III	62	46	14		32	28	
IV	34	28	6		27	7	
Lymph node metastasis							
Positive	147	119	28	0.000^**^	99	48	0.000^**^
Negative	96	53	43		42	54	

^a^Chi-square test, *P < 0.05, **P < 0.01

^b^American Joint Committee on Cancer (AJCC), patients were staged in accordance with the 7th Edition of the AJCC Cancer’s’ TNM Classification; *N of cases* number of cases, *T stage* tumor stage; *TNM* tumor node metastasis, *H* high, *L* low

**Table 3:** Univariate and multivariate analysis of overall survival in PDAC patients (*n* = 243)

Variables	Characteristics	Univariate analysis			Multivariate analysis		
		HR	95% CI	*P* value	HR	95% CI	*P* value
Sex	Male (ref) Female	1.080	0.825–1.412	0.576			
Age	< 60 (ref) ≥ 60	1.000	0.767–1.304	0.998			
Differentiation	Poor (ref)			0.009^**^			0.292
	Moderate	1.667	1.160–0.396	0.006	1.344	0.914–1.976	0.133
	Well	1.105	0.794–1.537	0.553	1.096	0.781–1.538	0.595
T stage	T1 (ref)			0.000^***^			0.005^**^
	T2	0.232	0.081–0.665	0.007	0.342	0.086–1.361	0.128
	T3	0.186	0.108–0.323	0.000	0.531	0.270–1.044	0.067
	T4	0.275	0.186–0.408	0.000	0.430	0.268–0.689	0.000
TNM stage	I (ref)			0.000^***^			0.000^***^
	II	0.175	0.095–0.321	0.000	0.154	0.067–0.357	0.000
	III	0.265	0.178–0.395	0.000	0.348	0.219–0.552	0.000
	IV	0.461	0.299–0.710	0.000	0.440	0.279–0.695	0.000
Lymph node metastasis	Positive (ref) Negative	3.190	2.362–4.307	0.000^***^	2.151	1.553–2.980	0.000^***^
SATB-1 expression	High (ref) Low	4.866	3.399–6.967	0.000^***^	3.473	2.305–5.235	0.000^***^
SDF-1 expression	High (ref) Low	3.429	2.564–4.585	0.000^***^	2.967	2.169–4.060	0.000^***^

*HR* hazard ratio, *95% CI* confidence interval, *TNM* tumor node metastasis, *T stage* tumor stage, *ref* reference

Cox regression analysis, Method: Enter, *p < 0.05, **p *<* 0.01, ***p *<* 0.001

**Table 4:** The relationship between SDF-1 expression and SATB-1 expression in pancreatic cancer tissues

		SATB-1 expression		Total
		Low	High	
SDF-1 expression	Low	49	53	102
	High	22	119	141
Total		71	172	243

Pearson *χ*^2^, *p* < 0.001

## Discussion

PDAC is one of malignant tumors with aggressive progression and high mortality rate. As a scirrhous carcinoma, abundant stromal content is one of PDAC hallmark features. CAFs play an important role in the tumor microenvironment, which has been proven to contain an autocrine-paracrine communication loop that impels tumor growth and metastasis^33–35^. In this study, we demonstrated that SDF-1, also known as C-X-C motif chemokine 12 (CXCL12), secreted by CAFs upregulated SATB-1 expression in pancreatic cancer cells via the SDF-1/CXCR4 axis. Then, we found that SDF-1-induced SATB-1 upregulation inhibits proliferation, migration, and invasion of pancreatic cancer cells, and conditioned medium from SW1990 and PANC-1 cells expressing SATB-1 maintains the properties of older CAFs, forming a SATB-1-centered positive feedback loop. Furthermore, we found that the expression of SATB-1 participates in the process of gemcitabine resistance. Finally, we tested the clinical correlations in human pancreatic cancer specimens. Our results revealed that the SDF-1/CXCR4/SATB-1 axis is vital to acceleration of the malignant progression and the gemcitabine resistance of pancreatic cancer cells and is associated with poor prognosis in PDAC patients.

Generally, CAFs are considered as positive mediators in the tumor microenvironment by secreting cytokines, chemokines, and proangiogenic factors. These mainly include, but not limited to, SDF-1, TGF-β1, heat shock factor 1, HGF, IL-6, tumor necrosis factor (TNF), and C-C motif chemokine ligand 2^7^. SDF-1 and TGF-β1 are two of the most-powerful and widely investigated molecules in various types of solid tumors, including pancreatic cancer^31,36,37^. Our data also indicated CAFs highly secreted SDF-1 compared with NAFs, which specifically binds to CXCR4 in pancreatic cancer cells and subsequently upregulates the expression of SATB-1 in pancreatic cancer cells.

SATB-1 is a nuclear matrix attachment region-binding protein located on chromosome 3p23, which regulates the global gene transcription and expression^38^. Since 2008, many researches gradually revealed that SATB-1 plays a crucial role in various types of malignant cancers, including breast cancer, laryngeal squamous cell carcinoma^39^, hepatocellular carcinoma^18^, colorectal cancer^40,41^, and gastric cancer^42^. However, the clinical and biological function of SATB-1 in pancreatic cancer deserves further investigation, especially in the tumor environment. As reported in our study, we found that SATB-1 is overexpressed in PDAC tissues and pancreatic cancer cell lines, and SATB-1 is upregulated by CAFs-secreted SDF-1. SATB-1 upregulation is relative with poor prognosis, which is similar to the inclusion of Chen Z’s research^27^. Our clinical specimens IHC staining found that SATB-1 expression is relative with SDF-1 expression, suggesting SATB-1 expression in pancreatic cancer cells may be upregulated by SDF-1 derived from CAFs in the stromal. Besides, experimental models were utilized to validate the value of SATB-1 in pancreatic cancer, which mainly included the proliferation, migration, and invasion of pancreatic cancer cells in vitro. Further research, however, is needed to explore the molecular mechanism of SATB-1 effect on cancer cell behavior.

At early stages of tumorigenesis, carcinoma in situ already involves “reactive” tumor fibrosis^7^. The recruitment of fibroblasts to tumor is largely governed by growth factors secreted by cancer cells and infiltrating immune cells, including TGF-β, PDGF and fibroblast growth factor 2^43^. Although the function of CAFs is debated, they are generally viewed as contributors to the process of cancer initiation and progression. How cancer cells maintain the properties of CAFs in the reciprocal loop between cancer cells and CAFs is unclear. In our study, we found that SATB-1 expression in pancreatic cancer cells mediated the maintenance of CAF properties and the activation of CAFs from NAFs. However, the paracrine-secreted factors and a precise molecular mechanism are currently not fully understood and deserve further investigation.

Gemcitabine resistance is a persistent clinical challenge for pancreatic cancer therapy. CAFs acted as a key player in promoting cancer cell evasion of chemotherapy drugs^44^. In this study, we revealed that the expression of SATB-1 mediated the gemcitabine sensibility of pancreatic cancer cells. SDF-1 has been revealed to take part in the development of chemotherapy resistance in various tumors, including breast cancer^45^, colorectal cancer^46^, glioblastoma^47^, acute myeloid leukemia^48^, and pancreatic cancer^49^. Feig et al.^50^ found that SDF-1 released by CAFs affected the anti-PD-1 immunotherapy. Our study emerged that CAF-promoted SATB-1 dysregulation in pancreatic cancer cells may contribute to gemcitabine resistance by some mechanism that requires further research.

In conclusion, we demonstrated that SDF-1-positive CAFs were significant in malignant progression and gemcitabine resistance, partially owing to paracrine induction of SATB-1 in pancreatic cancer cells. Overexpression of SATB-1 in pancreatic cancer cells was vital for the maintenance of CAF-like properties, thus forming a regulatory feedback loop in the tumor microenvironment. Our study provides the first evidence of the association of the SDF-1/CXCR4/SATB-1 axis with PDAC malignant progression and CAF maintenance, suggesting a potential new target of clinical interventions for pancreatic cancer patients.

## Materials and methods

### Patients and clinical samples

The 243 PDAC tissues were obtained from patients who underwent resection at Sun Yat-sen Memorial Hospital between 2006 and 2016. None of these patients received any preoperative chemotherapy or radiotherapy. The protocol was approved by the hospital’s Protection of Human Subjects Committee, and written informed consent was obtained from all patients before surgery. All samples were immediately snap-frozen in liquid nitrogen and stored at − 80 °C until further use. All samples were histologically diagnosed with PDAC by pathologic examination. The detailed clinicopathologic characteristics of patients are summarized in Table 1. The collection of follow-up data was carried out completely, and overall survival was defined as the time interval from the date of surgery to the date of death or the end-point of follow-up (October 2017).

### Isolation of pancreatic CAFs and paired NAFs

The isolation of stromal fibroblasts was previously described^11^. In brief, surgically resected pancreatic cancer tissues were obtained from four patients with pancreatic ductal adenocarcinoma. The written informed consents were obtained before operation. The fresh pancreatic tumor tissue and adjacent normal tissue (at least 2 cm from the outer tumor margin) were minced into 1–3 mm^3^ fragments and digested with 1 mg/ml collagenase I (#C0130, Sigma) at 37 °C for 2 h. The solution was centrifuged at 1000 rpm for 5 min, washed with phosphate-buffered saline twice and filtered with a 100-μm filter. Then, the isolated cells were seeded in a 10-cm dish. The details of the procedure can be found in the Supplementary Methods.

### Cell culture

Human pancreatic cancer cell lines (CFPAC-1, HPAC-1, PANC-1, Capan-2, MIAPaCa-2, SW1990, and BxPC-3) were purchased from the American Type Culture Collection (ATCC, Manassas, USA). The details of the procedure can be found in the Supplementary Methods.

### RNA extraction and qRT-PCR

Total RNA was extracted from frozen pancreatic cancer tissues and corresponding non-neoplastic tissues or cultured cell lines using TRIzol reagent (Invitrogen, Carlsbad, USA) according to the manufacturer’s instructions. The procedural steps were performed as described in the Supplementary Methods. All primer sequences for qRT-PCR are listed in Table S1 (see Additional file 1). For relative gene expression in tissues or cells, the levels were firstly normalized to β-actin expression as ΔCt and then compared with one of the tissues and converted to the fold change (2^−ΔΔCt^).

### Cell transfection and viral infection

For transient knockdown experiments, the siRNAs, CXCR4 siRNA (si-CXCR4), and synthetic sequence-scrambled siRNA (si-NC), were purchased from GenePharma Co. (Shanghai, China). The stable suppression of SATB-1 was performed by short hairpin RNA interference. All oligonucleotide sequences are provided in Table S2 (see Additional file 2). The transfection and infection procedures are described in the Supplementary Methods.

### Expression construct

The sequence of SATB-1 was synthesized and subcloned into pcDNA3.1 (Invitrogen, Shanghai, China). Ectopic expression of SATB-1 was achieved by using the pcDNA3.1-SATB-1 transfection, and an empty pcDNA vector (pcDNA3.1-NC) was used as the control. The expression level of SATB-1 was detected by western blot analysis.

### Half-maximal IC50 assay

Cells were collected and seeded in 96-well plates. The next day, fresh gemcitabine was added into cells at various concentrations of 0, 0.01, 0.1, 0.5, 1, 5, 10, 40, and 160 μM. After 72-h treatment, cells were photographed and quantified via CCK-8 assay. The half-maximal IC50 was calculated with the following formula: Ig(IC50) = Ig(Cmax)−Ig(Cmax/Csecond max) × (*P*−(3−*P*max−*P*min)/4), *P* was for the sum of all positive rates, *P*max was the maximal positive rate and *P*min was the minimum positive rate.

### Western blot analysis

Western blot assay was performed as described in the Supplementary Methods. Primary antibodies were: rabbit anti-human SATB-1 (1:1000, ab92307, Abcam), rabbit anti-human FAP (1:2000, ab28244, Abcam), rabbit anti-human α-SMA (1:1000, ab32575, Abcam), and rabbit anti-human GAPDH antibody (1:2500, CW0101, Cwbio, China). GAPDH was used as a loading control. Horseradish peroxidase-linked secondary antibody was goat anti-rabbit IgG (1:5000; Cwbio, China).

### ELISA

CAFs and matched NAFs (10^5^ cells/well) were seeded in six-well plates and cultured in Dulbecco's Modified Eagle's medium without serum for 3 days. Conditioned mediums were collected and detected for SDF-1 and TGF-β1 using ELISA kits (Cat: DSA00 and DB100B, R&D, USA) according to the manufacturer’s instructions. Each experiment was repeated at least three times.

### Immunohistochemical analysis

Paraffin-embedded samples of primary carcinomas were immunostained with primary rabbit anti-human SATB-1 (1:100, ab92307, Abcam), rabbit anti-human α-SMA (1:100, ab32575, Abcam), and rabbit anti-human SDF-1 (1:500, ab9797, Abcam) and Ki-67 (1:500, ab6526, Abcam) antibodies. The expression levels of SATB-1 and SDF-1 were scored semi-quantitatively, based on staining intensity and distribution, using the immunoreactive score as described elsewhere. Staining was assessed by two pathologists under double-blind conditions according to the scoring criteria. These procedures are described in the Supplementary Methods.

### Tumor formation assay

The athymic BALB/c nude mice (4–6 weeks old) were used for the tumor formation assay. SW1990 cells (2 × 10^6^) were injected subcutaneously into left and right bilateral hind legs of mice. Tumor volume was calculated as follows: Volume = (*L* × *W*^2^)/2 (*V*, volume; *L*, length diameter; *W*, width diameter). The animal care and experimental protocols were approved by the institutional guidelines of Guangzhou Province and by the Use Committee for Animal Care. All necessary steps were taken to minimize the suffering and distress caused to the mice. The procedures of tumor formation assay are described in the Supplementary Methods.

### Statistical analysis

All statistical analyses were performed using SPSS Statistics 18.0 software (IBM Chicago, IL, USA). The chi-square test (*χ*^2^-test) for nonparametric variables, and the two-tailed Student’s *t* test or one-way analysis of variance for parametric variables were used. All data are presented as the mean ± SD from at least three independent experiments, unless otherwise noted. Differences in patient survival were assessed using the Kaplan–Meier method and analyzed using the log-rank test in a univariate analysis. Univariate and multivariate Cox regression analyses were performed to assess the relative risk for each factor. All tests were two-sided, and results with *p* < 0.05 were considered statistically significant.

## Electronic supplementary material

Supplementary table 1

Supplementary table 2

Supplementary figure 1

Supplementary figure 2

Supplementary figure 3

Supplementary figure 4

Supplementary figure 5

Supplementary figure 6

Supplementary Materials and Methods

supplementary figure legends
