# Correction: Cancer-associated fibroblasts promote progression and gemcitabine resistance via the SDF-1/SATB-1 pathway in pancreatic cancer

**DOI:** 10.1038/s41419-021-03420-5

**Published:** 2021-03-03

**Authors:** Lusheng Wei, Huilin Ye, Guolin Li, Yuanting Lu, Quanbo Zhou, Shangyou Zheng, Qing Lin, Yimin Liu, Zhihua Li, Rufu Chen

**Affiliations:** 1 Guangdong Provincial Key Laboratory of Malignant Tumor Epigenetics and Gene Regulation, Sun Yat-sen Memorial Hospital, Sun Yat-sen University, Guangzhou, Guangdong Province China; 2 Department of Pancreatobiliary Surgery, Sun Yat-sen Memorial Hospital, Sun Yat-sen University, Guangzhou, Guangdong Province China; 3 Department of Radiology, Guangzhou Women and Children’s Medical Center, Guangzhou Medical University, Guangzhou, Guangdong Province China; 4 Department of Radiotherapy, Sun Yat-sen Memorial Hospital, Sun Yat-sen University, Guangzhou, Guangdong Province China; 5 Department of Medical Oncology, Sun Yat-sen Memorial Hospital, Sun Yat-sen University, Guangzhou, Guangdong Province China

Correction to: *Cell Death & Disease*

10.1038/s41419-018-1104-x published online 18 October 2018

Since online publication of this article, the authors noticed errors in Figs. 6C and 9A. In 6C an incorrect image was used for the primary image of “CAF-P4 FAP”. In Fig. 9A, incorrect brightness and scale were used for the SDF-1 ‘Low’ and ‘High’ images, respectively. The authors apologise for these errors. The corrected images are provided below.

**Figure:**
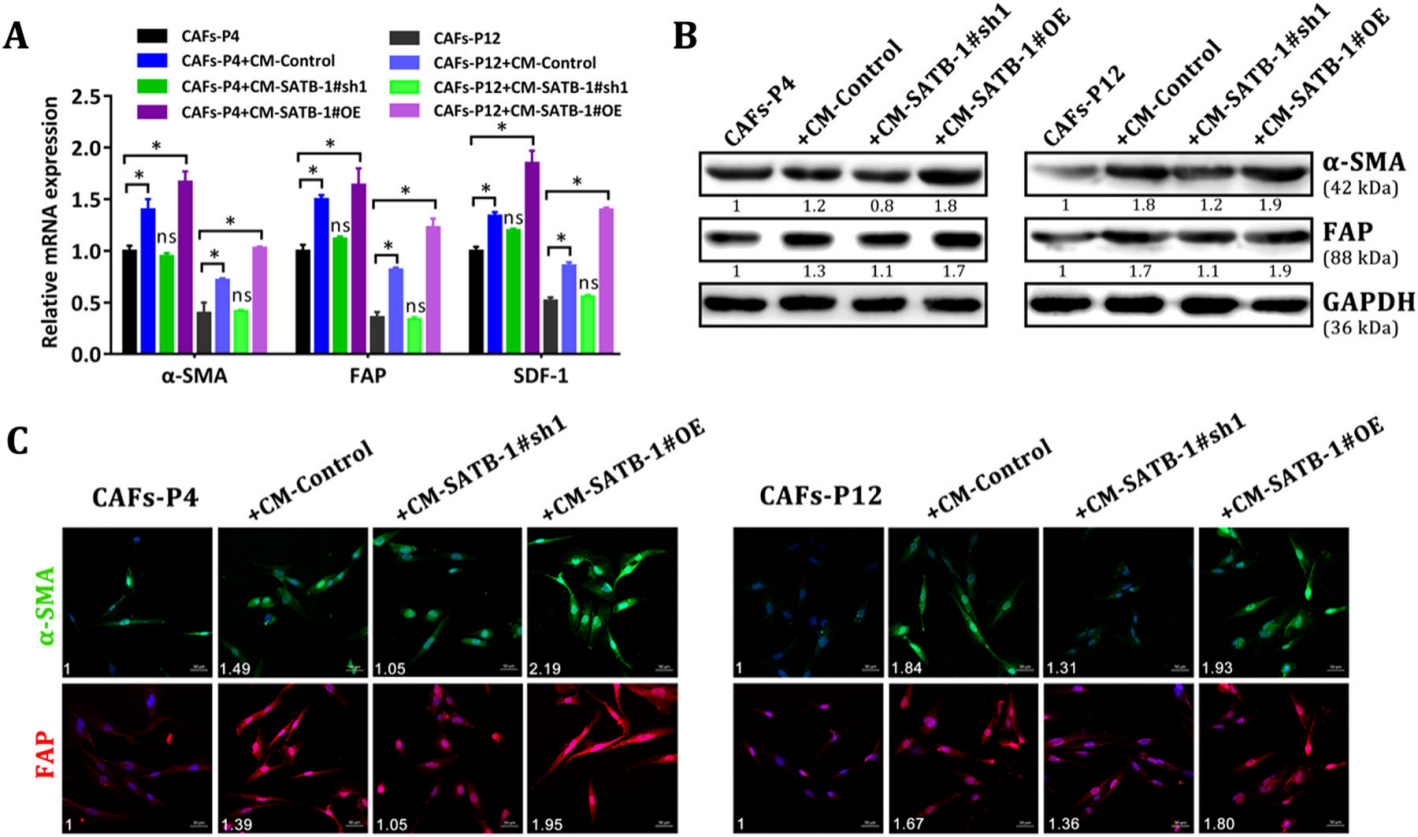
Fig. 6.

**Figure:**
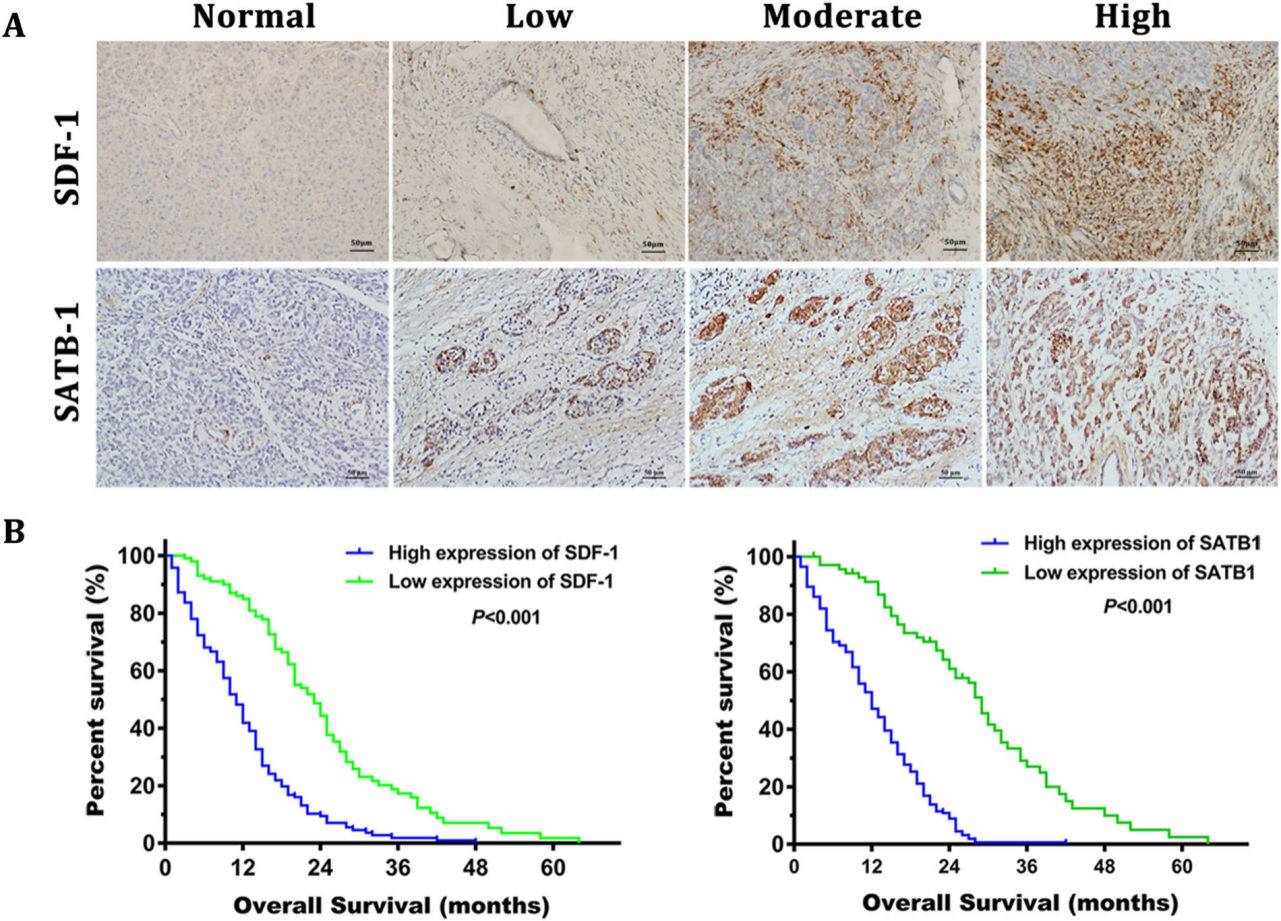
Fig. 9.

