# Supplementary material for: Cancer-associated fibroblasts promote progression and gemcitabine resistance via the SDF-1/SATB-1 pathway in pancreatic cancer
Source: Cell Death Dis. 2018 Oct 18;9(11):1065. doi: 10.1038/s41419-018-1104-x (PMC6194073; doi:10.1038/s41419-018-1104-x)
Supplement: Supplementary file 1 — Supplementary table 1 [file 41419_2018_1104_MOESM1_ESM.docx]

**Table S1. Primer sequences for qRT-PCR**

| primer | Forward sequence (5’ to 3’) | Reverse sequence (5’ to 3’) |
| --- | --- | --- |
| α-SMA | CCTTGAGAAGAGTTACGAGTTGC | ATGATGCTGTTGTAGGTGGTTT |
| FAP | AACCTCATCTCCCACGGCTTC | TTCTGTGCTTCTCCTCTTTGTCACT |
| FSP1 | CTTCCTGGGCTGCTTATCTGG | GTACTCGGGCAAAGAGGGTGA |
| SATB-1 | CTGTTACGCTGGAAAGAAGA | GCTGAGGAAGACTGAGGAA |
| CXCR4 | GGCCCTCAAGACCACAGTC | TTAGCTGGAGTGAAAACTTG |
| SDF-1 | CCCGAAGCTAAAGTGGATTC | TTCAGAGCTGGGCTCCTACT |
| VEGF | CCTGGTGGACATCTTCCAGGAGTA | CTCACCGCCTCGGCTTGTCACA |
| HGF | TACTGCAGACCAATGTGCTA | GAATTTGTGCCGGTGTGGTG |
| bFGF | GCGAATTCATGGCCGCCGGGAGCATCAC | CGCTCGAGTCAGCTCTTAGCAGACATTG |
| PDGF-A | GATACCTCGCCCATGTTCTG | TGGCACTTGACTGCTCGT |
| TGF-β1 | AACATGATCGTGCGCTCTGCAAGTGCAGC | AAGGAATAGTGCAGACAGGCAGG |
| IGF-1 | CTAGGCACTCTGCTTGC | CTTGGGCATGTCAGTGTGGC |
| IL-6 | ATGAACTCCTTCTCCACAAGC | CTACATTTGCCGAAGAGCCCTCAGGCTGGACTG |
| CCL18 | AAACTCGAGCTGCCCAGCATCATGAAGG | TTTGGATCCCCTCAGGCATTCAGCTTCAG |
| TNF-α | ATGAGCACTGAAAGCATGATC | TCACAGGGCAATGATCCCAAAGTAGACCTGCCC |
