# Supplementary material for: Cancer-associated fibroblasts promote progression and gemcitabine resistance via the SDF-1/SATB-1 pathway in pancreatic cancer
Source: Cell Death Dis. 2018 Oct 18;9(11):1065. doi: 10.1038/s41419-018-1104-x (PMC6194073; doi:10.1038/s41419-018-1104-x)
Supplement: Supplementary file 2 — Supplementary table 2 [file 41419_2018_1104_MOESM2_ESM.docx]

**Table S2. Oligonucleotide sequences**

| **Gene** | **Forward sequence (5’ to 3’)** | **Reverse sequence (5’ to 3’)** |
| --- | --- | --- |
| si-CXCR4 | GGAUCAGCAUCGACUCCUUTT | AAGGAGUCGAUGCUGAUCCTT |
| si-NC | UUCUCCGAACGUGUCACGUTT | ACGUGACACGUUCGGAGAATT |
| sh-SATB-1#1 | CCGGGGTCGATGTGGCAGAATATAACTCGAGTTATATTCTGCCACATCGACCTTTTTG | AATTCAAAAAAGGAGCTGCCACATCGACCACTCGAGTTTGCCACATCGACCGCCTTCCTT |
| sh-SATB-1#2 | CCGGAGCTGAAAGAGACCGAATATACTCGAGTATATTCGGTCTCTTTCAGCTTTTTTG | AATTCAAAAAAGGAGGGTCTCTTTCAGCTAACTCGAGTTGGTCTCTTTCAGCTCTTCCTT |
| sh-NC | CCGGTTCTCCGAACGTGTCACGTAACTCGAGTTACGTGACACGTTCGGAGAATTTTTG | AATTCAAAAAATTCTCCGAACGTGTCACGTAACTCGAGTTACGTGACACGTTCGGAGAA |
