# Supplementary figures and images for: Cancer-associated fibroblasts promote progression and gemcitabine resistance via the SDF-1/SATB-1 pathway in pancreatic cancer
Source: Cell Death Dis. 2018 Oct 18;9(11):1065. doi: 10.1038/s41419-018-1104-x (PMC6194073; doi:10.1038/s41419-018-1104-x)

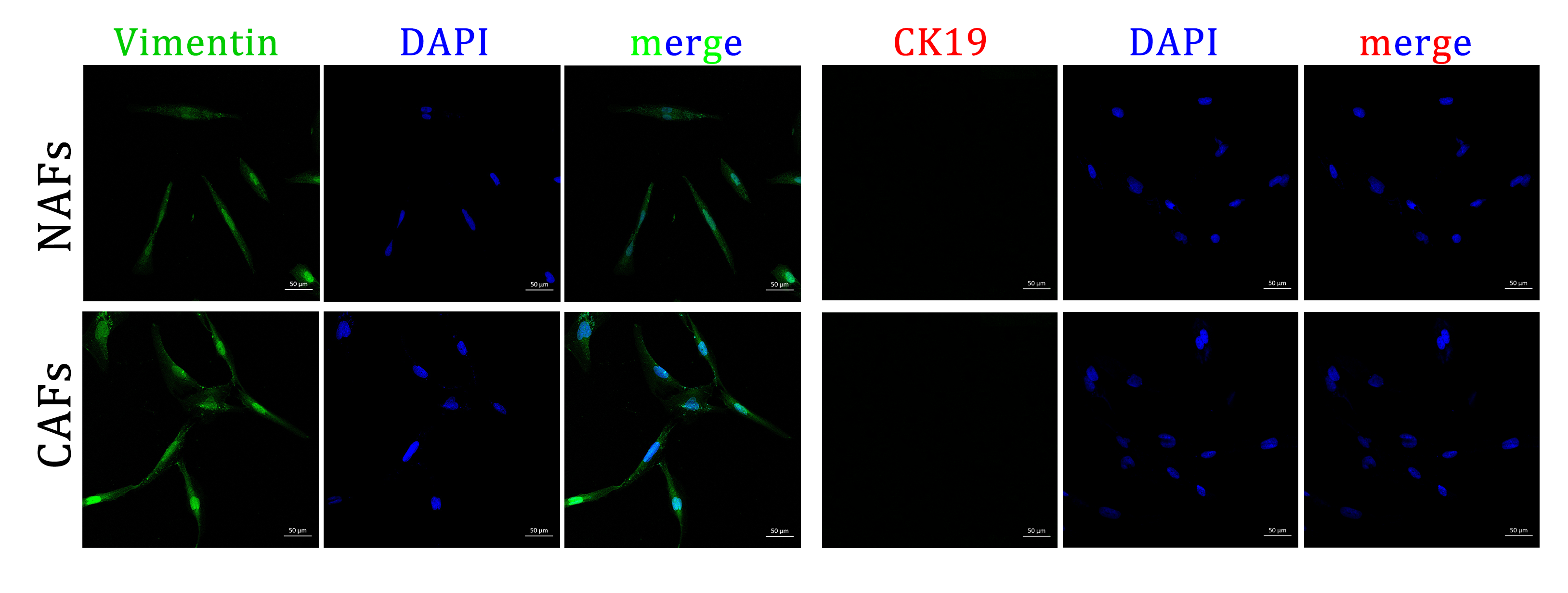

Supplement: Supplementary file 3 — Supplementary figure 1 [file 41419_2018_1104_MOESM3_ESM.tif]

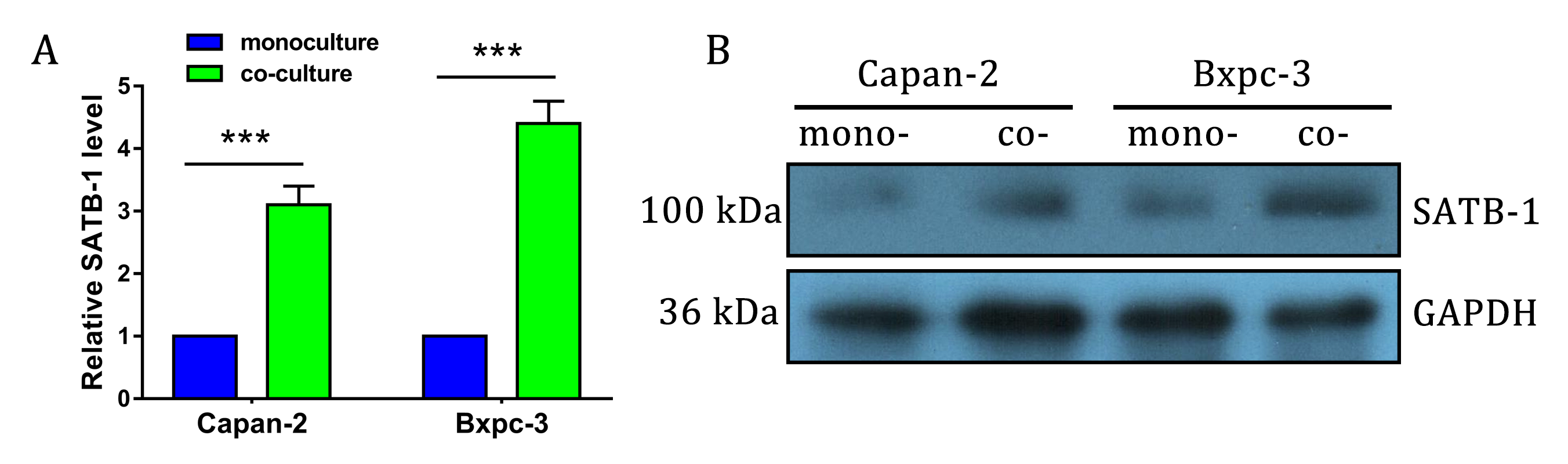

Supplement: Supplementary file 4 — Supplementary figure 2 [file 41419_2018_1104_MOESM4_ESM.tif]

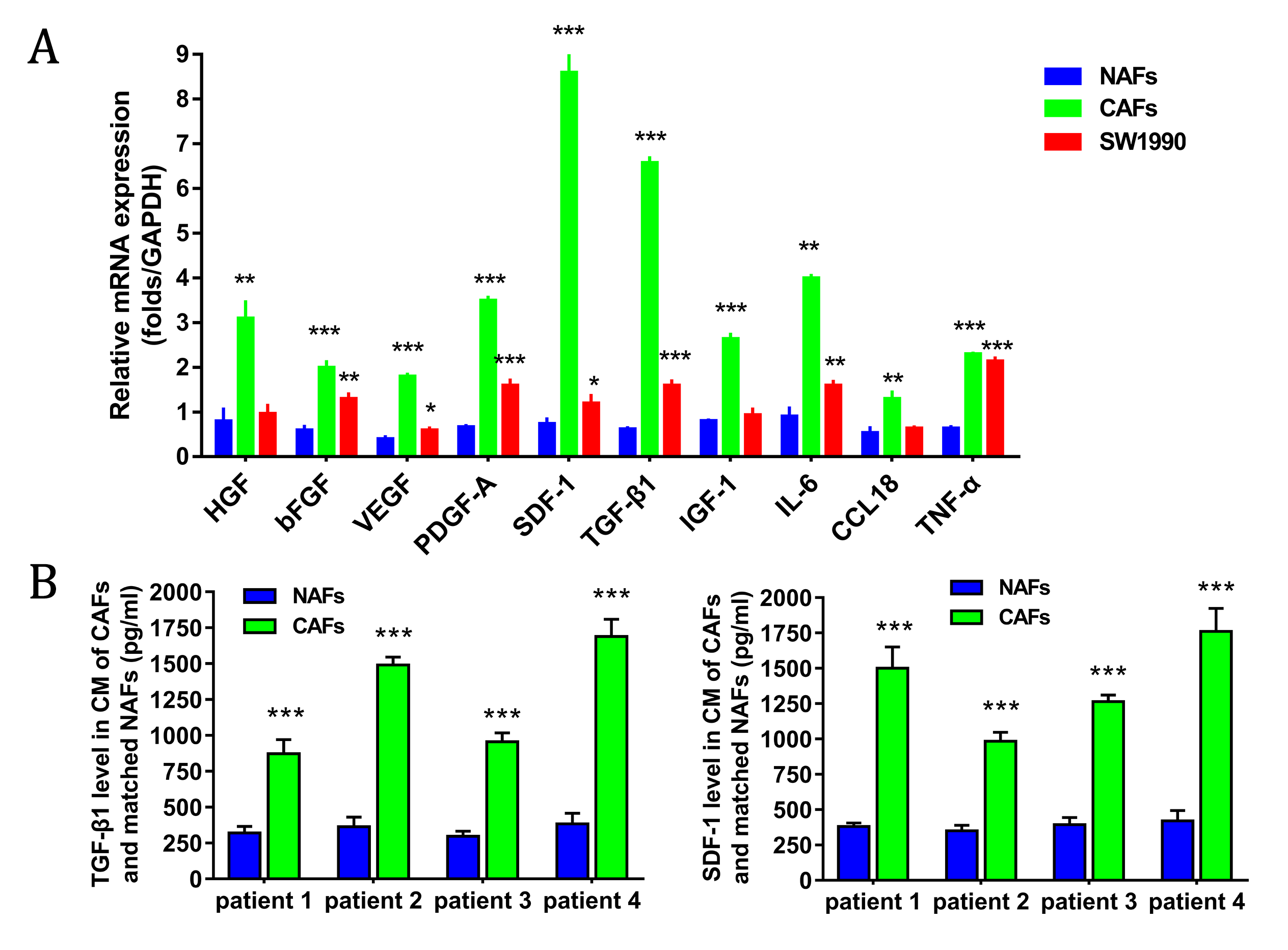

Supplement: Supplementary file 5 — Supplementary figure 3 [file 41419_2018_1104_MOESM5_ESM.tif]

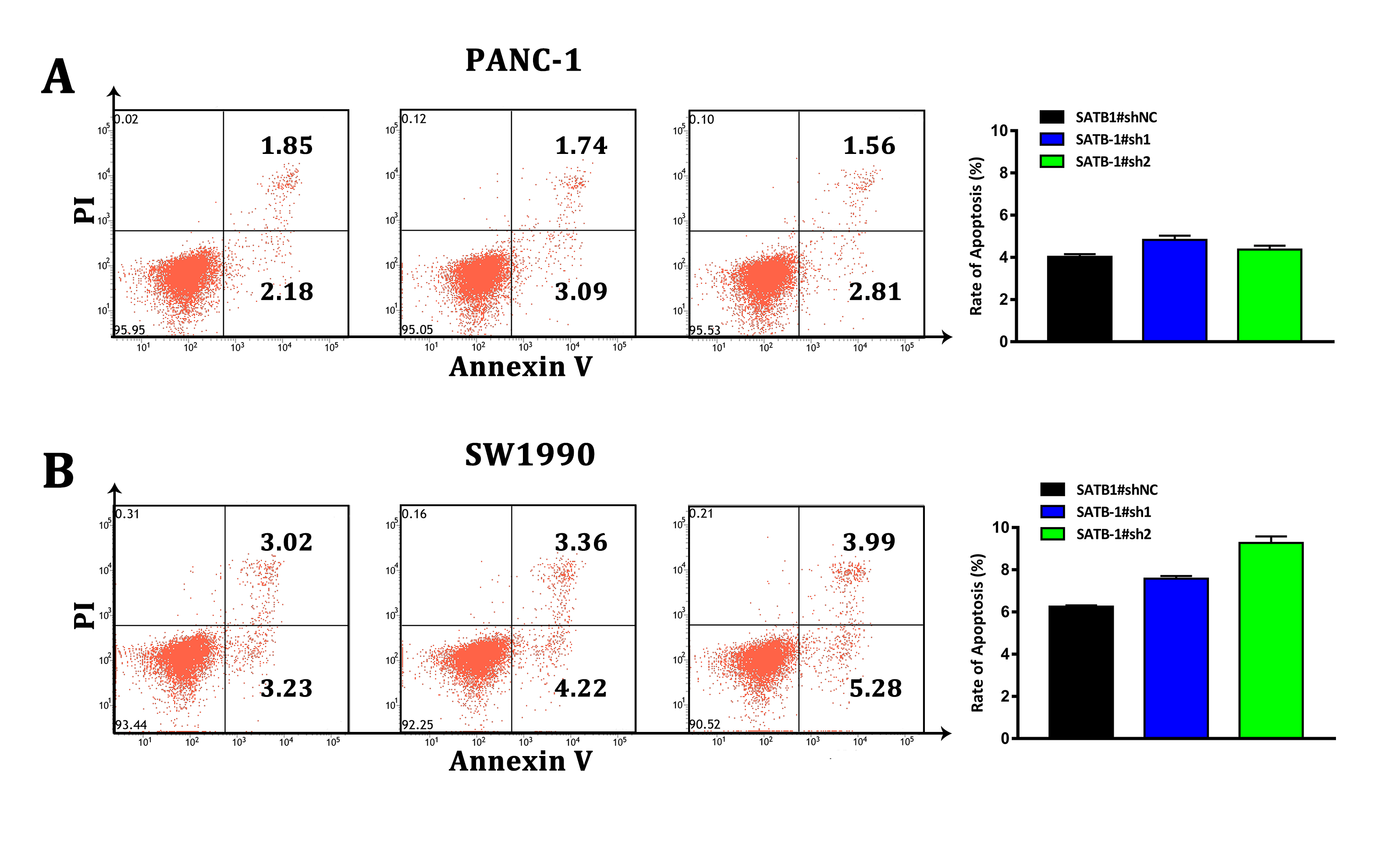

Supplement: Supplementary file 6 — Supplementary figure 4 [file 41419_2018_1104_MOESM6_ESM.tif]

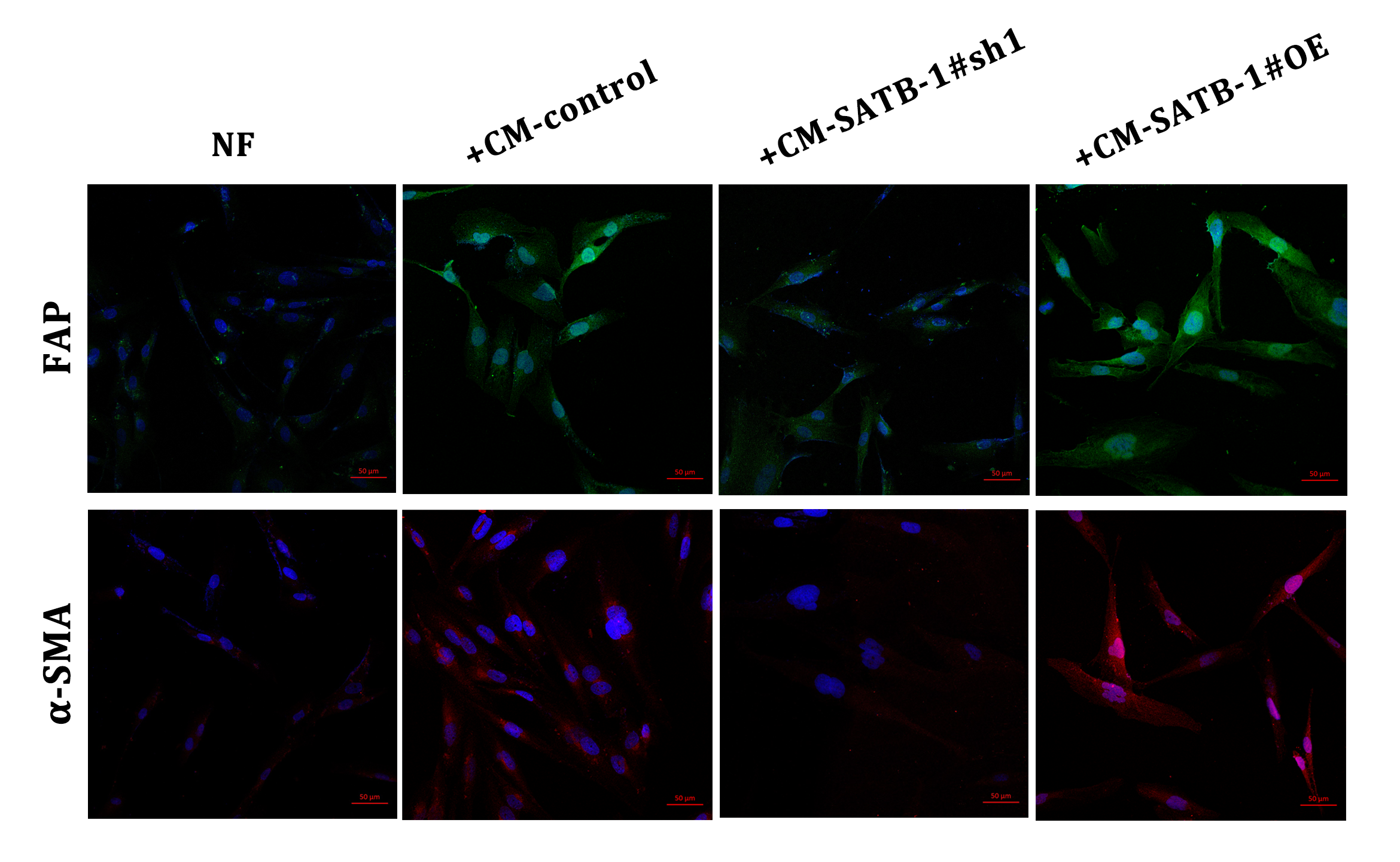

Supplement: Supplementary file 7 — Supplementary figure 5 [file 41419_2018_1104_MOESM7_ESM.tif]

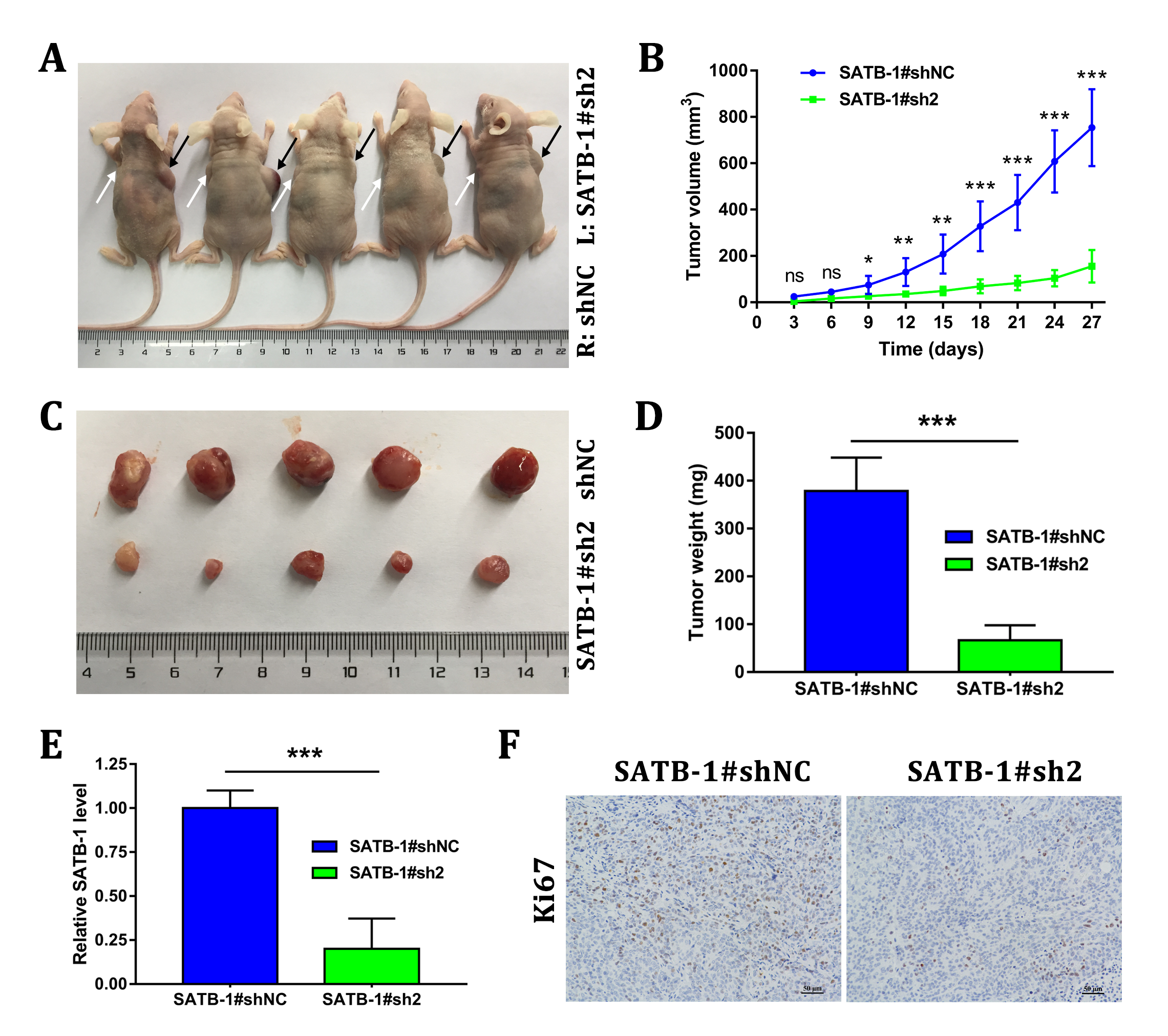

Supplement: Supplementary file 8 — Supplementary figure 6 [file 41419_2018_1104_MOESM8_ESM.tif]
