# Supplementary material for: Cancer-associated fibroblasts promote progression and gemcitabine resistance via the SDF-1/SATB-1 pathway in pancreatic cancer
Source: Cell Death Dis. 2018 Oct 18;9(11):1065. doi: 10.1038/s41419-018-1104-x (PMC6194073; doi:10.1038/s41419-018-1104-x)
Supplement: Supplementary file 9 — Supplementary Materials and Methods [file 41419_2018_1104_MOESM9_ESM.docx]

**Supplement Materials and Methods**

**Isolation of pancreatic CAF and paired NF**

The isolation of stromal fibroblasts was based on what previously described ^53^. Briefly, surgically resected pancreatic cancer tissue were obtained from four patients with pancreatic ductal adenocarcinoma. The written informed consents were obtained before operation. The fresh pancreatic tumor tissue and adjacent normal tissue (at least 2 cm far away from the outer tumor margin) were minced into 1-3 mm^3^ fragments and digested with 1 mg/ml Collagenase I (#C0130, Sigma) at 37℃ for 2 hours. The collagenase was dissolved with DMEM containing 10% fetal bovine serum (GIBCO) and 3% penicillin/streptomycin (GIBCO). The resulting solutions containing the cells and smaller fragments were centrifuged at 1000 rpm for 5 min. The pellets were washed with PBS twice and filtered with a 100-μm filter. Then, the isolated cells were seeded in a 10 cm dish with DMEM/F12 containing 10% fetal bovine serum, 100 U/ml penicillin, and 100 mg/ml streptomycin at 37℃ in a humidified atmosphere containing 5% CO_2_. The culture medium was changed twice a week. After the fibroblasts were sub-cultured for 3 passages, the cultures were free of contamination of the epithelial cells. All primary fibroblasts used for this study were between passages 3 to passages 8.

**Cell culture**

Human pancreatic cancer cell lines (CFPAC-1, HPAC-1, AsPC-1, PANC-1, Capan-2, MIA PaCa-2, SW1990 and BxPC-3) and the immortal human pancreatic duct epithelial cell line (HPDE6-C7) were purchased from the American Type Culture Collection (ATCC, Manassas, USA). Cells were maintained in Dulbecco’s modified Eagle’s medium (DMEM; GIBCO-BRL; Invitrogen, CA, USA) or Roswell Park Memorial Institute medium (RPMI 1640; GIBCO-BRL; Invitrogen, CA, USA) supplemented with 10% fetal bovine serum (FBS; GIBCO-BRL; Invitrogen, CA, USA), 100 U/ml penicillin, and 100 mg/ml streptomycin and cultured at 37˚C in humidified air with 5% CO_2_. CFPAC-1 and SW1990 were cultured with recombinant human SDF-1alpha (Cat. No. 96-300-28A, Pepro Tech, USA) at 5, 10 and 20 μg/ml. Selected co-cultures or conditioned mediums from CAFs were treated with neutralizing antibodies to SDF-1 (Cat. No. ab9797, Abcam, Cambridge, MA) at 4 μg/ml. NFs were cultured with recombinant human TGFβ1 (Cat. No. 96-AF-100-21C, Pepro Tech, USA) at 10 μg/ml. Selected co-cultures or conditioned mediums from pancreatic cancer cells were treated with neutralizing antibodies to TGF-β1 (Cat. No. ab64715, Abcam, Cambridge, MA) at 2 μg/ml.

For cell condition medium, the conditioned medium was collected after cells were cultured with complete medium for 24 h, followed by centrifugation at 3000 rpm for 10 min. The condition medium was filtered with a 0.22μm-sterile filter and then stored in a -80 ℃ refrigerator for further usage.

**RNA extraction and quantitative real-time PCR (qRT-PCR)**

Total RNA was extracted from tissues or cultured cell lines using TRIzol reagent (Takara, 9108, Japan) according to the manufacturer's instructions. RNA concentration was measured with NanoDrop ND-2000 spectrophotometers (Life Technologies, CA, USA) and then the total RNA (500 ng) was transcribed to cDNA in a final volume of 10 μl using Prime Script^TM^ RT Master Mix (Takara, RR036A, Japan). Real-time PCR was performed using SYSB^®^ Premix Ex Taq^TM^ Ⅱ (Takara, RR820A, Japan) and analyzed on a Roche Light-Cycler system (Roche, Basel, Switzerland). GAPDH was used as an internal control to normalize mRNA levels. All the primer sequences were listed in Table S1. The qRT-PCR data were analyzed and expressed relative to CT (cycle threshold) values. The qRT-PCR results were analyzed and showed as the fold change (2^-∆∆CT^). For expressions in tissues, the levels were firstly normalized to GAPDH expression as △CT and then compared with one of the tissues and converted to the fold change (2^-∆∆CT^). For the analysis of relative gene expressions in tissues or cells, the levels were compared with the controls and converted to the fold change (2^-∆∆CT^). The quantitative PCR reaction for each sample was performed in triplicate.

**Cell transfection and viral infection**

For transient knockdown experiments, the following small interfering RNAs (siRNAs) were as follows: SATB1 siRNA (si-SATB1), CXCR4 siRNA (si-CXCR4) and synthetic sequence-scrambled siRNA (si-NC) were purchased from GenePharma Co. (Shanghai, China). The siRNAs were transfected into cells using Lipofectamine 3000 (Life Technologies, CA, USA) following the manufacturer’s instruction. After 48 h, the efficiency of siRNA knockdown was confirmed via qRT-PCR and Western blot analysis. For stable knockdown experiments, SATB1 shRNA (sh-SATB1) and scrambled control RNA (sh-NC) were inserted into the lentiviral vector (pLKO.1-puro vector). 72 h after transfection of 293T cells, the viral supernatants were collected. Using a LentiX™ Concentrator overnight at 4°C (Clontech, Mountain View, CA, USA), lentiviral particles were concentrated and titered to 10^9^ TU/ml (transfection unit/ml). SW1990 cells (5 × 10^5^ cells/well) were seeded in 6-well culture plates and maintained in DMEM with 10% FBS and then infected with virus and polybrene 24 h later. Positive clones were screened with puromycin (2μg/ml and 5μg/ml, respectively) for 2-3 weeks to establish the following new stable cell lines: SW1990-sh-SATB1 (SATB1 stable knockdown) and SW1990-sh-NC (negative control). The same transfection was performed into PANC-1 cells. All oligonucleotide sequences were listed in Table S2.

**Cell proliferation assay**

Cell proliferation was measured using a cell counting kit-8 (CCK-8) assay according to the manufacturer’s instructions. Cells were seeded in 96-wells plates (4 × 10^3^ cells/well), at the appropriate time (24, 48, 72 and 96 h), CCK-8 solution (10 μL, Dojindo Molecular Technologies, Kyushu, Japan) was added and the cells were incubated for 2h at 37^o^C. Absorbance was measured at a wavelength of 450 nm. The assays were repeated three times.

**Cell apoptosis and cell-cycle analysis**

For cell apoptosis, SW1990 and CFPAC-1 (5 × 10^5^ cells) were collected via trypsin digestion, washed with cold phosphate-buffered saline (PBS), and resuspended with 400 μl binding buffer. Half of cells were added with 5 µl Annexin V-FITC (Becton Dickinson Biosciences, USA) and incubated away from light for 15 min at room temperature. Then, 10 µl PI (Becton Dickinson Biosciences, USA) was added into cells and the control (the rest half of cells). Cells were detected and analyzed using a BD FAC Scan Flow Cytometer (BD, Mountain View, USA) in 30 min.

For cell-cycle analysis, SW1990, CFPAC-1 and PANC-1 cells (5 × 10^5^ cells) were collected, washed with PBS and fixed with 70% ice-cold ethanol at -20℃ for 24h at least. Cells were rewashed with PBS and incubated with staining solution containing with 500 μg/mL propidium iodide and 100 μg/mL RNaseA for 30 min. Then, cells were analyzed by flow cytometry. Each study was repeated at least three times.

**Half maximal inhibitory concentration (IC50) assay**

Cells were collected and seeded in 96-wells plates. The next day, the freshly gemcitabine was added into cells at various concentrations of 0, 0.01, 0.1, 0.5, 1, 5, 10, 40 and 160 μM. After 72 h treatment, cells were photograghed and detected via CCK-8 assay. The half maximal inhibitory concentration (IC50) was calculated with the following formula: Ig(IC50)=Ig(Cmax)-Ig(Cmax/Csecond max)×(P-(3-Pmax-Pmin)/4), P was for the sum of all the positive rate, Pmax was for the maximal positive rate and Pmin was for the minimum positive rate.

**Wound healing assay and transwell assay**

After pre-treatment, cells were seeded in 6-well plates and cultured until 90% confluency. A wound was scratched with a 10-ul sterile pipette tip on cell monolayer. Then, cells were washed and exchanged with medium containing 1% FBS cultured for 48 h. To prevent cell proliferation, mitomycin C (10 μg/ml) was added into the medium for 1 hour. At different time points (0 h, 24h, 48h), photographs of the wounds were taken to evaluated the area healed by migrated cells. Image Pro Plus was used for analysis of wound-healing capability. Experiments were performed at least three times.

Transwell chambers (24-well insert, 8 μm, Corning Costar Corp, USA) were performed to evaluate the migration and invasion capability of pancreatic cancer cells. CAFs were seeded in the lower chamber of 24-well plates with 1×10^4^ cells per well 24 hours ago or not. After pre-treatment as above, cells (2.5×10^4^ cells) in 100 μl serum-free DMEM were inoculated in the upper chamber, coated with (invasion) or without (migration) 1:8 diluted Matrigel (BD Biosciences, NJ, USA). DMEM supplement with 10% FBS were added into the lower chamber. After incubation for 24 hours at 37°C , the migrated or invaded cells were fixed with 4% paraformaldehyde and stained with crystal violet solution, and the cells on the upper surface of the chamber membrane were wiped with a cotton swab. The migrated or invaded cells were counted in five randomized high power fields under a microscope. Experiments were performed at least three times.

**Cell immunofluorescence staining**

Cells were seeded and cultured in 24-well plates with cell glass cover slips. After treatment, cells were washed with PBS twice and fixed with 4% paraformaldehyde for 30 minutes. Then, cells were treated with 0.3% Triton-X 100 and blocked with 5% BSA at room temperature for 30 minutes, followed by overnight incubation at 4°C with primary antibodies: FAP (1:200, ab28244, Abcam), α-SMA (1:200, ab32575, Abcam). After washing with PBS, cells were incubated with the Alexa Fluor 488 goat anti ribbit antibody (Invitrogen, USA) for 1 h at room temperature. Cells were then washed three times with PBS and mounted in Vectashield mounting medium containing DAPI (ab104139, Abcam). The slides were analyzed using a confocal laser scan microscope.

**Western blot analysis**

Cells were harvested and the lysates were prepared by extraction proteins with RIPA lysis buffer (Beyotime, China) supplemented with a protease inhibitor cocktail (Roche, Pleasanton, CA, USA) and PMSF (Roche). The protein concentration was calculated using a bicinchoninic acid protein assay kit (Beyotime, China). Equivalent amounts of proteins (35 μg) from each sample were separated by 10% sodium dodecyl sulfatepolyacrylamide gel electrophoresis (SDS) and then transferred to PVDF membranes. The membranes were blocked in 5% non-fat dried milk for 1 h at room temperature and then incubated overnight at 4℃with the following primary antibodies: rabbit anti-human SATB1 (1:1000, ab92307, Abcam), rabbit anti-human FAP (1:2000, ab28244, Abcam), rabbit anti-human α-SMA (1: 1000, ab32575, Abcam), and rabbit anti-human GAPDH antibody (1:2500, CW0101, Cwbio, China). After washing with TBST, the membranes were incubated with horseradish peroxidase (HRP)-conjugated secondary antibodies (1:5000, Cwbio, China) at room temperature for 1 h. An ECL chemiluminescence kit (Millipore, WBKLS0100) was used to visualize the specific blots and autoradiograms were quantified by densitometry (Quantity One software, Bio-Rad, Hercules, CA, USA) using GAPDH.

**Immunohistochemical staining and scoring**

Paraffin-embedded samples of primary carcinomas were stained for SATB1 or SDF-1. Sections were deparaffinized in xylene and rehydrated in a graded series of ethanol, followed by heat-induced epitope retrieval. Antigen retrieval was performed in Tris-EDTA solution (pH = 9.0) in a microwave oven for 15 min. Then, 3% hydrogen peroxide was used to block the activity of endogenous peroxidases for 15 min at room temperature. After washed with PBS, the sections were incubated with primary antibodies overnight at 4°C as following: rabbit anti-human SATB1 (1:100, ab92307, Abcam), rabbit anti-human α-SMA (1: 100, ab32575, Abcam), and rabbit anti-human SDF-1 (1: 500, ab9797, Abcam) and Ki-67 (1:500, ab6526, Abcam). After washing three times in PBS, sections were incubated with goat anti-rabbit HRP-conjugated secondary antibody (PV-6001, ZSGB-bio, China) for 30 min at 37℃, followed by staining with 3, 3’-diaminobenzidine (as the color reagent) and hematoxylin (as a counterstain for nuclei). All sections were dehydrated in increasing concentrations of ethanol and xylene and mounted with neutral gum. Normal goat serum and PBS substituting the primary antibody were used as negative controls. Images were visualized using a Nikon ECLIPSE Ti (Fukasawa, Japan) microscope system and processed with Nikon software.

The expression levels of SATB1 and SDF-1 were scored semiquantitatively based on staining intensity and distribution using the immunoreactive score (IRS) as described elsewhere ^54,55^. Briefly, immunoreactive score (IRS) = SI (staining intensity) × PP (percentage of positive cells). SI was assigned as following: 0 = negative; 1 = weak; 2 = moderate; 3 = strong. The percentage of positive tumor cells were calculated after at least 10 view fields at 400 × magnification were counted per section. PP is defined as 0 = 0%; 1 = 0-25%; 2 = 25-50%; 3 = 50-75%; 4 = 75-100%. For categorization of the continuous SATB1 and SDF-1 values into low and high, we chose a commonly used cutoff point for the measurements (range 0-12: low, cut point of 0-4; high, cut point of 6-12). Staining was assessed by two pathologists under double-blind conditions according to the scoring criteria. Cases with discrepancies were jointly reevaluated until a consensus was reached.

**Tumor formation assay in a nude mouse model**

The athymic BALB/c nude mice (4-6 weeks old) were purchased and maintained at the Laboratory Animal Center of Sun Yat-sen University in a pathenogen-free environment under controlled humidity and temperature, with a 12-hours light-dark cycle. Mice were given continuous access of food and water. The animal care and experimental protocols were approved by the institutional guidelines of Guangdong Province and by the Ethical and Welfare Committee of Sun Yat-sen University. SW1990 cells stably transfected with SATB-1- sh1, SATB-1-sh or sh-NC were cultured and collected. A total of 100 μl of 2×10^6^ suspended cells with stable knockdown of SATB1, or mock cells, were respectively injected subcutaneously into left and right bilateral hind leg of mice. At day 3 after the injection of tumor cells, the tumor growth was evaluated once every 3 days by measuring the length and the width with electronic calipers. The tumor volume was calculated using the following formula: V = (L × W^2^)/2 (V, volume; L, length diameter; W, width diameter). The mice were sacrificed by cervical dislocation at 28 days post injection, and tumors were collected for further study (weight measurement and RNA extraction). SATB1 levels were determined by qRT-PCR and Western blot analysis.

**Statisitcal analysis**

Data are presented as the mean±S.D. of three independent experiments. All statistical analyses were performed with SPSS 23.0 (SPSS Inc, Chicago, IL, USA). The chi-square test (*χ*^2^ test) for non-parametric variables, and Student’s *t* test or one-way analysis of variance (ANOVA) for parametric variables was used (two-tailed). Cumulative survival time was calculated using the Kaplan–Meier method and analysed by the log-rank test. A multivariate Cox-proportional hazards model was used to estimate the adjusted hazard ratios and 95% confidence intervals and to identify independent prognostic factors. The threshold for statistical significance was set at ^*^*P* < 0.05, ^**^*P* < 0.01.

**References:**

^1^ Richards, K. E. *et al.*, Cancer-associated fibroblast exosomes regulate survival and proliferation of pancreatic cancer cells. *ONCOGENE* **36** 1770 (2017).

^2^ Berns, S., Uzelac, S., Gonzalez, C. & Jaeger, J., Methodological considerations of measuring disability in bipolar disorder: validity of the Multidimensional Scale of Independent Functioning. *BIPOLAR DISORD* **9** 3 (2007).

^3^ Mack, K. F., Leinung, M., Stieve, M., Lenarz, T. & Schwab, B., Clinical feasibility test on a minimally invasive laser therapy system in microsurgery of nerves. *Minim Invasive Ther Allied Technol* **17** 292 (2008).
