# Supplementary material for: Cancer-associated fibroblasts promote progression and gemcitabine resistance via the SDF-1/SATB-1 pathway in pancreatic cancer
Source: Cell Death Dis. 2018 Oct 18;9(11):1065. doi: 10.1038/s41419-018-1104-x (PMC6194073; doi:10.1038/s41419-018-1104-x)
Supplement: Supplementary file 10 — supplementary figure legends [file 41419_2018_1104_MOESM10_ESM.docx]

**Supplementary figure legends**

**Figure S1. Isolated NAFs and CAFs both expressed vimentin but not CK19.** Immunofluorescence staining was used to identify the mesothelial origin with vimentin and the epithelial origin with CK19.

**Figure S2. CAFs upregulated the expression of SATB-1 in Capan-2 and BxPC-3 cells.** qRT-PCR (A) and western blot (B) were used to analyze the mRNA and protein level in Capan-2 and BxPC-3 cells co-cultured with CAFs. n=3 (replicating from 3 patients), ^***^ for *p*<0.001.

**Figure S3. SDF-1 was highly secreted by CAFs compared with NAFs.** (A) The mRNA expression of ten well-known cellular factors (HGF, bFGF, VEGF, PDGF-A, SDF-1, TGF-β1, IGF-1 IL-6 CCL18 and TNF-α) were analyzed with qRT-PCR in NAFs, CAFs and SW1990 cells. n=3(replicating from 3 patients), ^***^ for *p*<0.001. (B-C) ELISA analysis was used to detect the secretion level of SDF-1 and TGF-β1 in NAFs and CAFs. n=3(replicating from the same patient), ^***^ for *p*<0.001.

**Figure S4.** (**A-B**) FACS analysis shows the apoptosis rates of PANC-1 and SW1990 cells with SATB-1 knockdown.

**Figure S5. NAFs were activated into CAFs by conditioned mediums from cancer cells with SATB-1 expression.** NAFs were treated with conditioned medium from SW1990 with SATB-1#shNC or SATB-1#sh1, or Capan-2 with SATB-1 overexpression. After 3 days, the features of NAFs were detected with immunofluorescence staining. NAFs highly expressed FAP and α-SMA, showing the activated status.

**Figure S6. Knockdown of SATB-1 inhibits tumor growth in mouse xenograft models.** (A) SW1990 cells with a stable SATB-1 knockdown (sh2) or mock cells were subcutaneously implanted in nude mice. At 27 days after injection, SW1990 cells transfected with SATB-1#sh2 (white arrow) and mock cells (black arrow) developed primary tumors. (B) Tumor growth curve. The points and bars represent means±SD. n=5, ns: not significantly different. ^**^: p<0.01, ^***^: p<0.001. (C) The images of harvested tumors. (D) Tumor weights are shown as means±SD. n=5, ^***^: p<0.001. (E) The qRT-PCR assay analyzed the mRNA expression level of SATB-1 in tumor tissues from SATB-1#sh2 SW1990 cells compared with sh-NC SW1990 cells. (E) IHC staining showed the reduced expression of Ki-67 in tumor samples from SW1990-SATB-1#sh2 cells.
